# Supplementary material for: Isolating hydrogen in hexagonal boron nitride bubbles by a plasma treatment
Source: Nat Commun. 2019 Jun 27;10:2815. doi: 10.1038/s41467-019-10660-9 (PMC6597567; doi:10.1038/s41467-019-10660-9)
Supplement: Supplementary file 1 — Supplementary Information [file 41467_2019_10660_MOESM1_ESM.pdf]

Supplementary Information for:

Isolating hydrogen in hexagonal boron nitride bubbles by a plasma treatment

Li He, et al.

Supplementary Optical Images

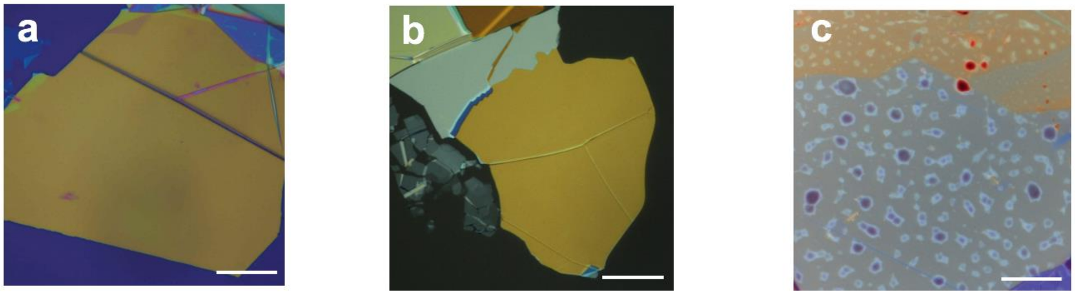

Supplementary Figure 1 | a-c, Optical images of *h*-BN flakes corresponding to the AFM height images shown Fig. 1(b-d), respectively. Scale bars: 20 μm.

Transmission Electron Microscopy (TEM) measurement

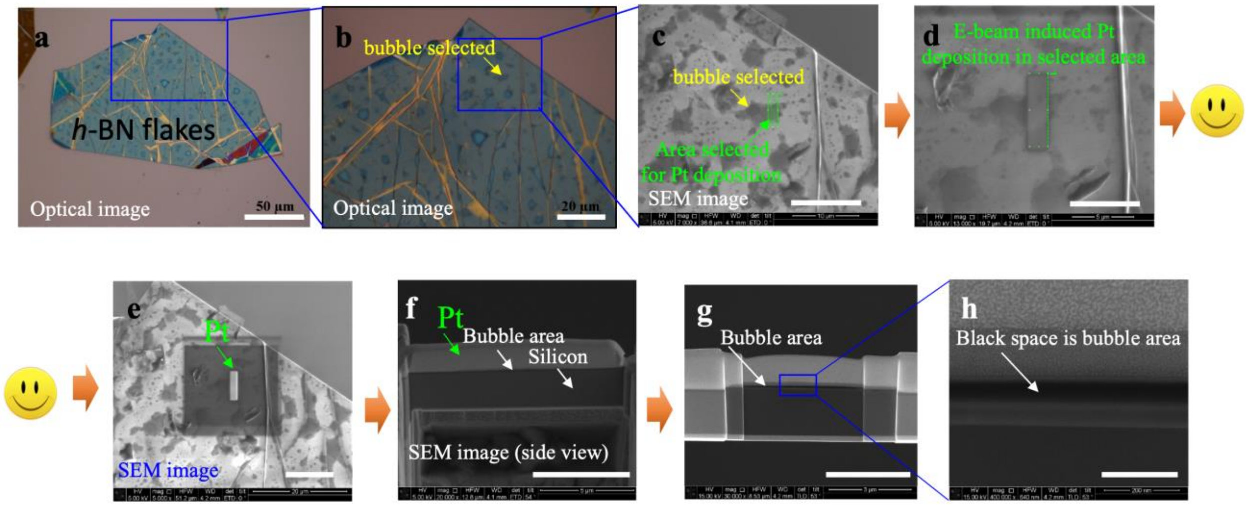

Supplementary Figure 2 | OM and SEM images acquired during TEM specimen preparation. a-b, Optical images of *h*-BN flakes with bubbles. The SEM image sequence **(c-h)** corresponds to the chronological steps required for the fabrication of a *h*-BN bubble TEM specimen. The specimen was prepared by dual beam system (Helios NanoLab 600). **c-e,** the SEM images of the top view of the *h*-BN flake with bubbles. **f-h,** Cross-sectional SEM image of the specimen after FIB milling. Scale bars: (a), 50 μm, (b), 20 μm, (c-e), 10 μm, (f), 5 μm, (g), 3 μm, (h), 200 nm.

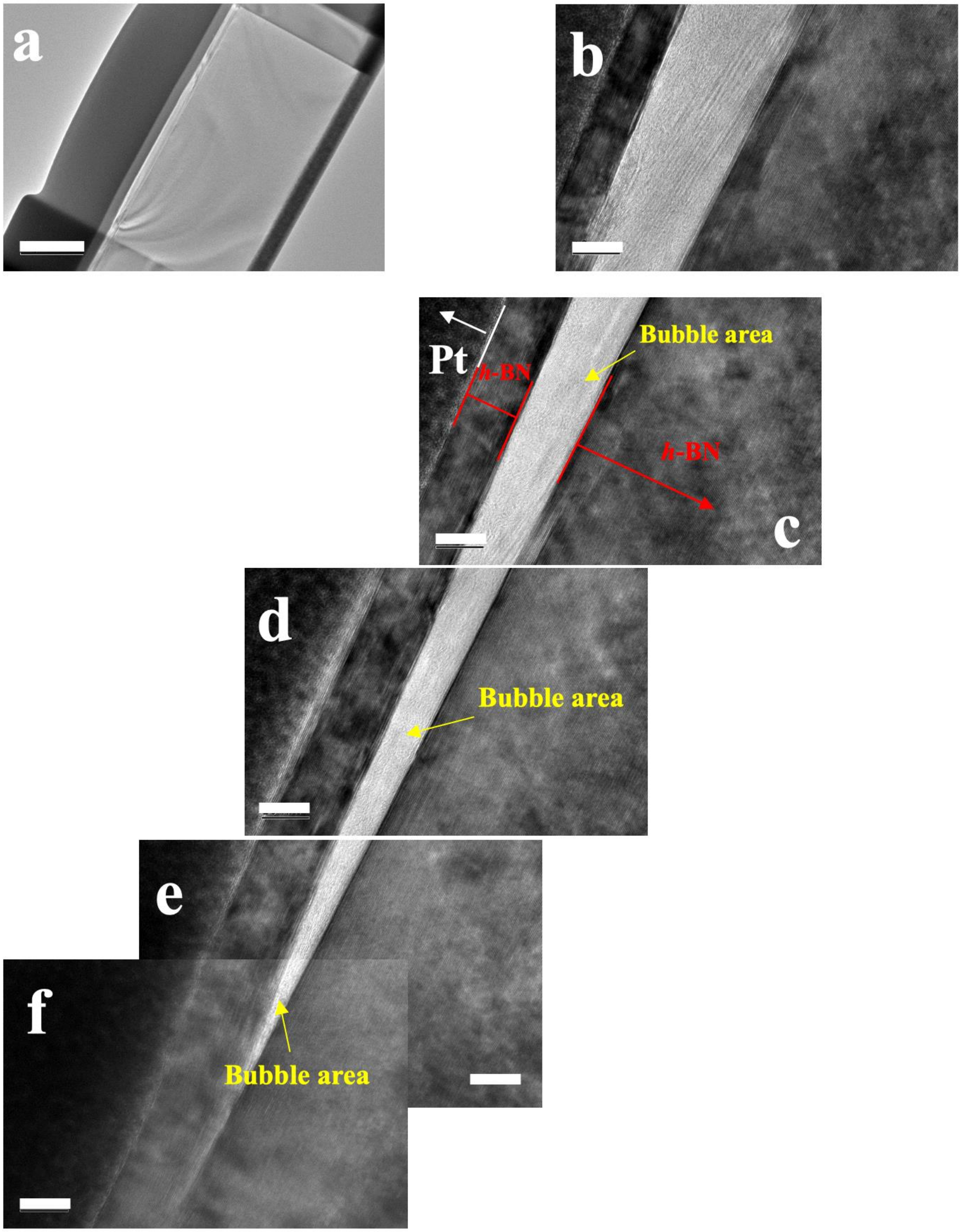

Supplementary Figure 3 | Enlarged cross-sectional TEM view of bubble position inside *h*-BN. a, Cross-sectional picture of the *h*-BN bubble-sample after FIB fabrication. Scale bar: 1 µm; **b-f,** Specific enlarged TEM images from middle to border area of *h*-BN bubble-position. Scale bars: 10 nm.

A group of cross sectional images measured by scanning electron microscope (SEM) and TEM are presented to characterize the *h*-BN bubble structure. The specimen for TEM measurement was prepared by Focused Ion Beam (FIB) system (Helios NanoLab 600) which is equipped with a function of SEM. Supplementary Fig. 2 shows the specific process to make a specimen of *h*-BN bubbles. The green rectangle indicated in Supplementary Fig. 2c is the area selected for e-beam induced Pt deposition. After Pt deposition (Supplementary Fig. 2e), the TEM specimen for bubble cross-sectional imaging was ready after FIB shaping (Supplementary Fig. 2f-g, Supplementary Fig. 3a). The SEM images are captured by the SEM in the FIB system. Then, the specimen was transferred to a TEM chamber (JEOL 2100F, operated at 200 kV). The enlarged cross-sectional scanning images by TEM are shown in a set of pictures which are manually combined to exhibit the *h*-BN bubble from the middle to the border area (Supplementary Fig. 3b-f). Note that the gap of the bubble is so narrow (in a few tens of nm) that we can hardly see it in the overall cross-sectional TEM image of Supplementary Fig. 3a.

X-ray photoelectron spectroscopy (XPS)

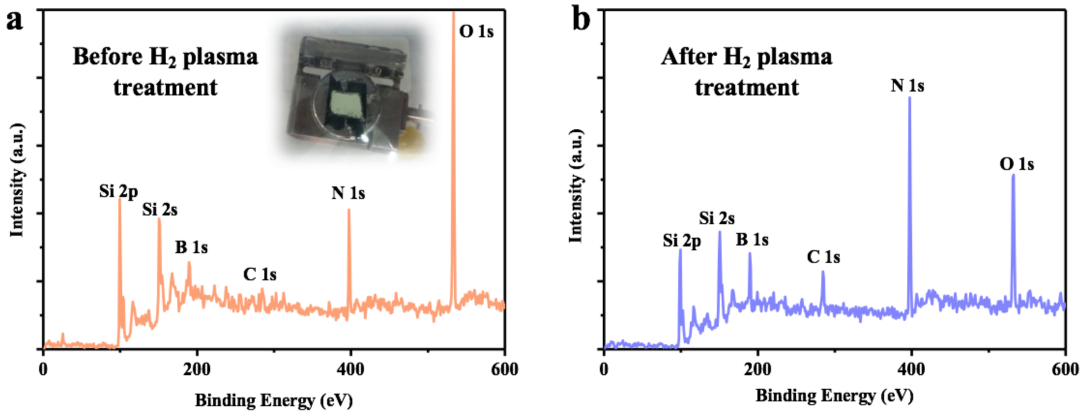

Supplementary Figure 4 | XPS survey analysis of *h*-BN flakes on quartz substrate before (a) and after (b) H_2 plasma treatment in the full energy range. The B 1s and N 1s peaks are visible. Inset shows a quartz substrate with *h*-BN flakes placed on a sample holder.

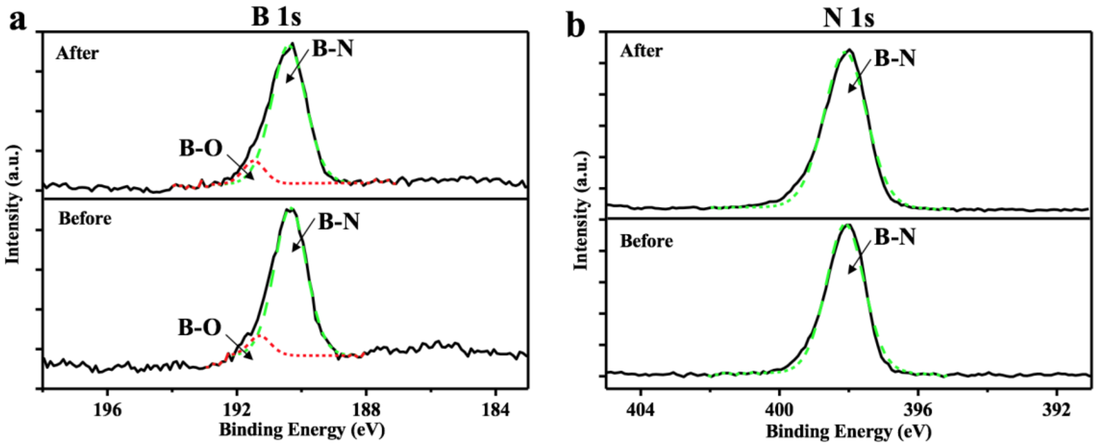

Supplementary Figure 5 | Fine XPS spectra survey of *h*-BN flakes before and after H_2 plasma treatment. B 1s **(a)** and N 1s **(b)** spectra obtained from *h*-BNs flakes. Dashed lines are fitting curves.

	B-N(FWHM) eV	B-O(FWHM) eV	N-1s eV
h-BN with bubbles (after plasma treatment)	190.4(1.24)	191.5(0.8)	398.1(1.44)
h-BN (before plasma treatment)	190.3(1.17)	191.3(0.8)	398.1(1.29)

Supplementary Table 1 | Peak position and FWHM of XPS spectra of the *h*-BN flakes before and after plasma treatment.

Firstly, X-ray photoelectron spectroscopy (XPS) measurement was carried out to investigate the influence of H_2 plasma treatment on *h*-BN flakes. Substrates with *h*-BN flakes are measured on SPECS XPS system using monochromatic Mg Kα line at a base pressure of 10^{-9} mbar before and after plasma treatment. The XPS survey analysis in the full energy range is shown in Supplementary Fig. 4. XPS spectra of *h*-BN flakes are calibrated with reference to C 1s at 284.5 eV. The specific measurement results are given in Supplementary Fig. 5. Supplementary Fig. 5a shows the narrow scan B 1s of *h*-BN before and after plasma treatment. As shown in Supplementary Fig. 5a, there is an obvious peak in the binding energy range from 184 eV to 196 eV, which can be fitted into two peaks. The main peak (the green dashed curve) corresponds to B-N bond of B 1s, while the tiny peak (the red dashed curve) corresponds to the B-O bond which were caused by annealing in O_2 flow at 600 ℃. Annealing in O_2 flow is necessary to remove tape residues after exfoliation. Supplementary Fig. 5b shows the N 1s core level spectra that were fitted to only one curve which corresponds to B-N bond. It is clear that the XPS spectra of *h*-BN flakes does not change obviously even after H_2 plasma treatment. As shown in Supplementary Table 1, the *h*-BN XPS peaks with bubbles have broadened, this phenomenon can be reasonably attributed to the *h*-BN expansion caused by bubbles formation.

Energy Dispersive X-Ray (EDX) analysis on elemental composition of *h*-BN

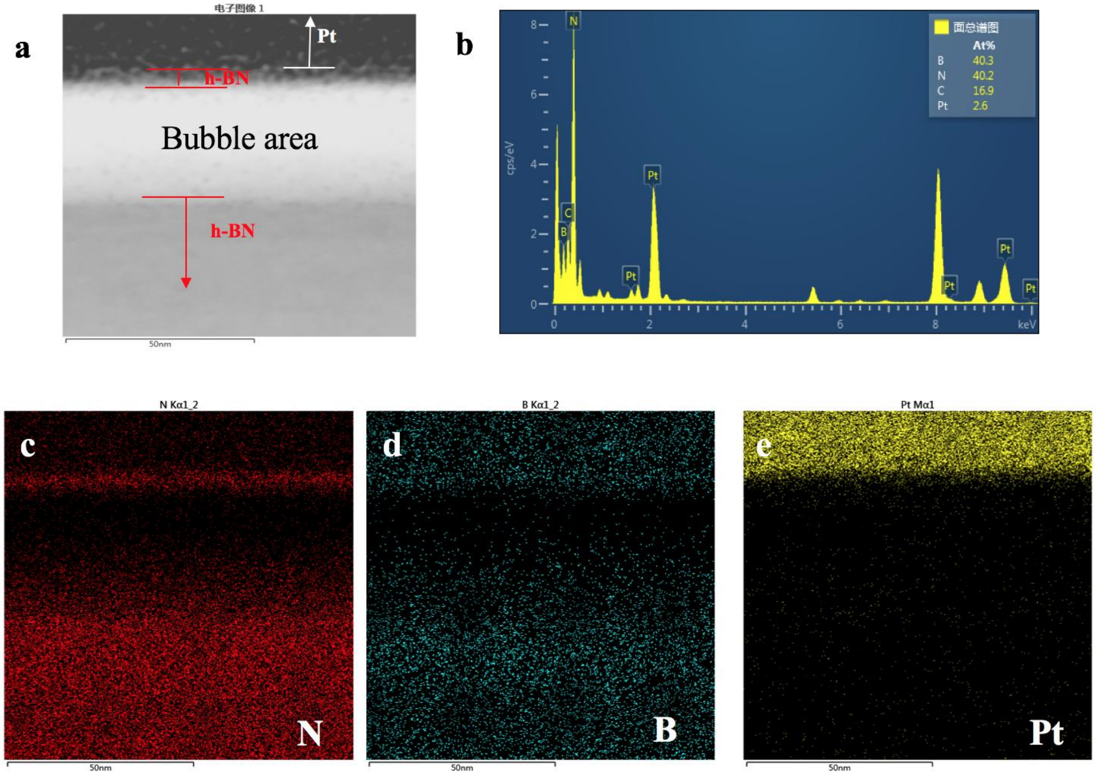

Supplementary Figure 6 | EDX elemental analysis of the cross-sectional interface of *h*-BN specimen. a, Scanning transmission electron microscope (STEM) image shows the selected EDX inspection area. *h*-BN and Pt area are indicated. **b,** The resulting spectrum and tabulated results reveal that B, N, and Pt are the main elements present with C element being mainly caused by hydrocarbon contamination. **c-e,** The corresponding elemental mapping of N, B and Pt, respectively. EDX elemental mapping indicates that the bubble area is empty.

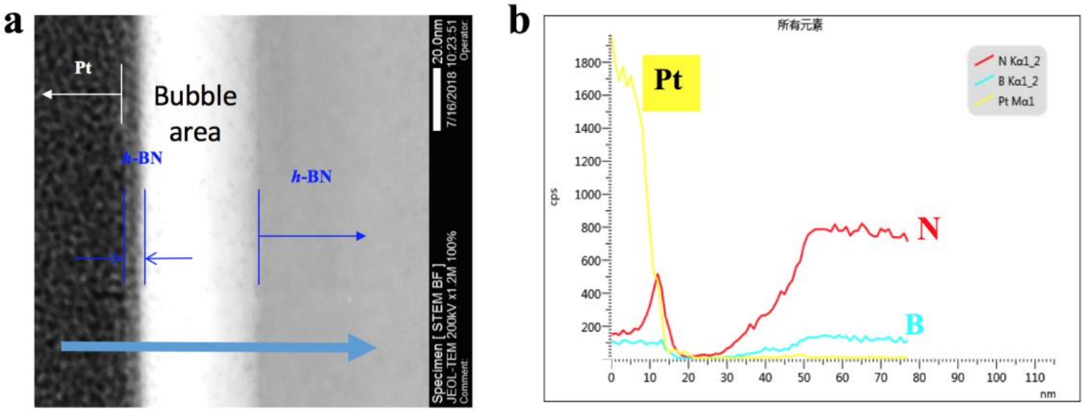

Supplementary Figure 7 | Element line profile scan for Pt, N and B using EDX on STEM. a, High-resolution STEM cross-sectional image of a *h*-BN bubble**. b,** The lateral distribution of Pt, N and B by using EDX analysis via line scan.

EDX is a semi-quantitative x-ray technique used to identify the elemental composition of materials. The scanning transmission electron microscope (STEM) is equipped with a EDX detector. Supplementary Fig. 6 shows EDX elemental analysis of the cross-sectional interface of *h*-BN specimen. As shown in Supplementary Fig. 6b, EDX spectrum shows peaks corresponding to the elements (B, N, C and Pt), where B and N the main elements. Elemental mapping of a sample and image analysis are given in Supplementary Fig. 6c-e. EDX elemental mapping clearly shows that the bubble area is empty.

Fourier-transform infrared (FTIR) spectra

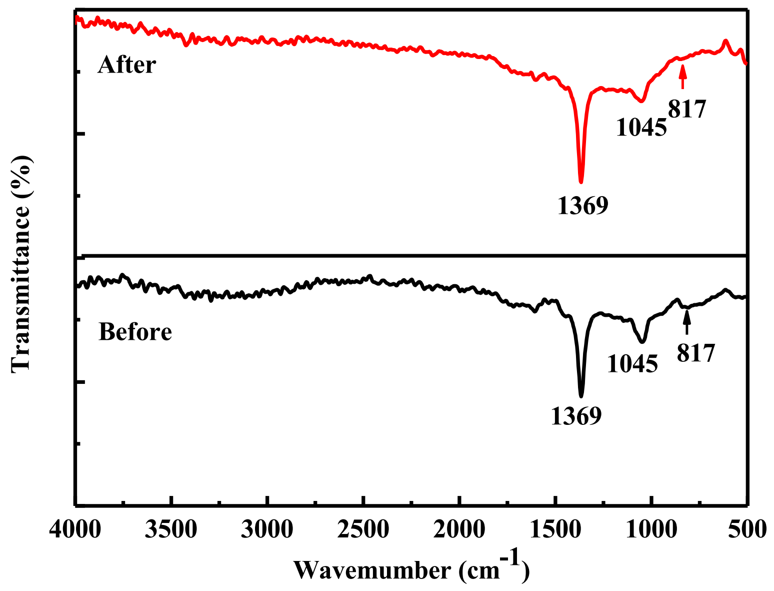

Supplementary Figure 8 | FTIR spectra of the *h*-BN flakes on silicon substrates before and after H_2 plasma treatment.

Hydrogen plasma treatment was carried out on *h*-BN flakes exfoliated on pristine <100> silicon substrate to produce bubbles. FTIR measurement was conducted at ~1 mbar (on Bruker IFS 66v/S) before and after hydrogen plasma treatment. The FTIR spectra of the *h*-BN before and after H_2 plasma treatment are shown in Supplementary Fig. 8. There are 3 obvious dips in the range from 500 to 4000 cm^{-1}. The dip at 1369 cm^{-1} was derived from the in-plane stretching vibration of *h*-BN, and the dip at 817cm^{-1} was owning to out-of-plane bending absorption of the B-N-B. The absorption peak appears at 1045 cm^{-1} was a fingerprint of the presence of Si-O bonds, which was derived from the substrate. The similar characteristics in FTIR spectra indicate that the plasma treatment did not chemically cause obvious change in *h*-BN flakes.

Mass spectra (MS) analysis

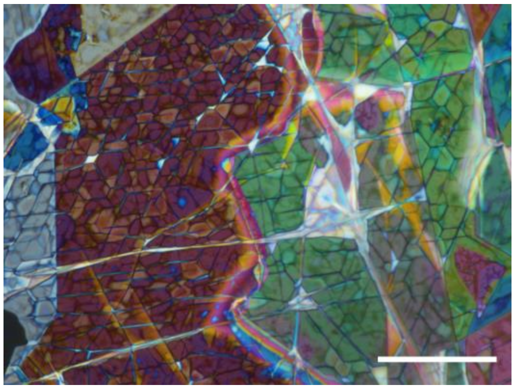

Supplementary Figure 9 | Optical microscope image of a *h*-BN flake with dense bubbles on a quartz substrate, which is the sample prepared for the mass spectra measurement. Scale bar indicates 20 μm.

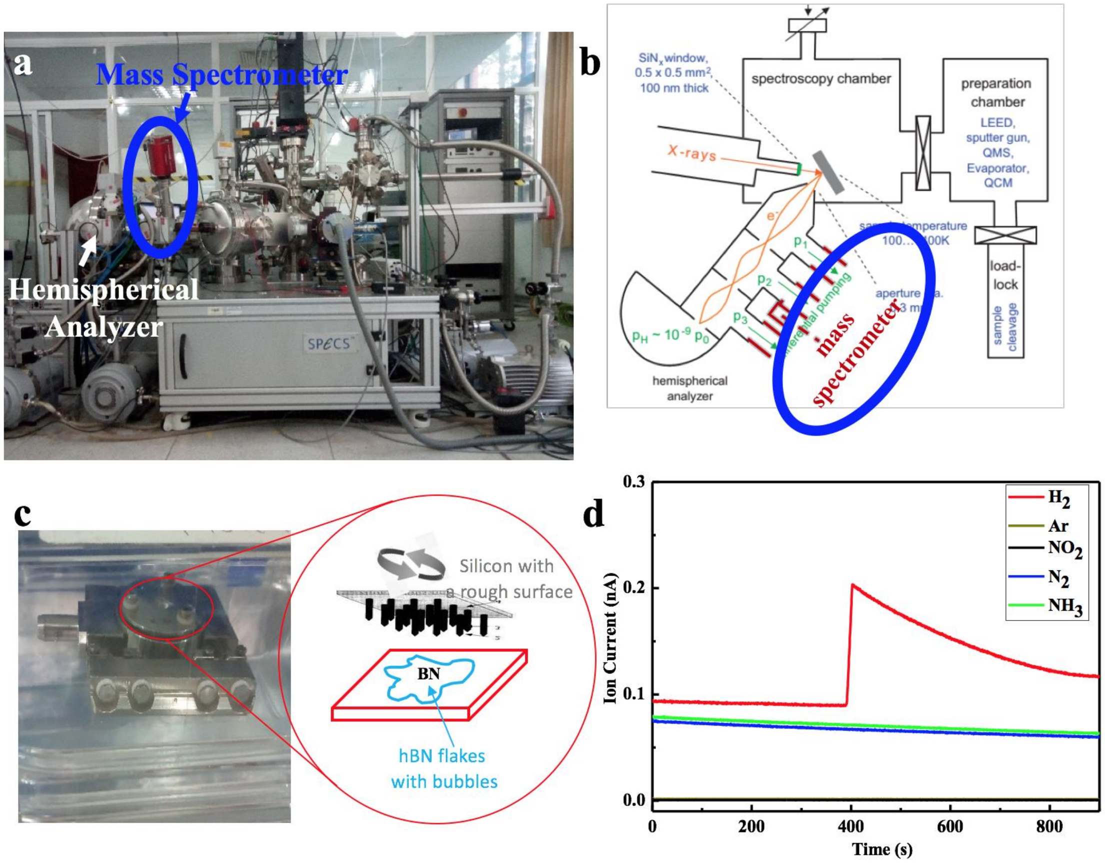

Supplementary Figure 10 | Experimental details about the mass spectra analysis on *h*-BN bubbles. a, A UHV system equipped with a mass spectrometer**. b,** Schematic for the vacuum system. **c,** sample holder used for the mass spectrum analysis. **d,** Real-time monitoring on different gases by mass spectrometer.

Mass spectrometry measurement can be conducted to analyze gases in the bubble. Mass spectra (MS) analysis was carried out on a UHV vacuum system with quadrupole mass spectrometer (Pfeiffer QMS 220 M). The sample for MS measurement is a quartz substrate in size of 10 × 10 mm with a high density of *h*-BN flakes, which has very dense bubbles (Supplementary Fig. 9). The measurement system is shown in Supplementary Fig. 10a and the schematic of its structure is given in Supplementary Fig. 10b. The substrate with *h*-BN bubbles were installed in a homemade sample holder (see Supplementary Fig. 10c). As shown in Supplementary Fig. 10c, another silicon substrate with rough surface were placed on the quartz substrate with *h*-BN bubbles surface, and both substrates were assembled on sample holder gently. The holder has a screw which can slide the top silicon substrate. The sliding can spoil the *h*-BN bubbles on underlying quartz substrate. The experiment was carried out in a UHV chamber with a base pressure of 10^{-9} mbar. After the base pressure in the chamber became stable, we rotated the screw to break *h*-BN bubbles. The mass spectrum variation was recorded and plotted in Supplementary Fig. 10d. We set several possible gases (H_2, Ar, NO_2, N_2 and NH_3) for monitoring. As shown in Supplementary Fig. 10d, only the hydrogen increased suddenly after the bubbles were broken, and then gradually decreases with time. This experiment proves that the gas inside the bubble is hydrogen.

Here, we discuss some details about the experimental design. The density of *h*-BN flakes on 10×10 mm quartz substrate is about 30×30 pieces, and the density of bubbles on each piece of *h*-BN flake is about 100×100. Assuming the pressure in a bubble is about 10 bar, and the volume of the bubble is about 10 μm × 10 μm × 0.1 μm. The volume of the chamber is 0.1 m^3. Even if 1% of bubbles were broken, the pressure will reach about 9×10^{-8} mbar. Compared with the base pressure of chamber, 10^{-9} mbar, the gas is sufficient to be detected.

Plane-view STEM measurement for a *h*-BN multilayer

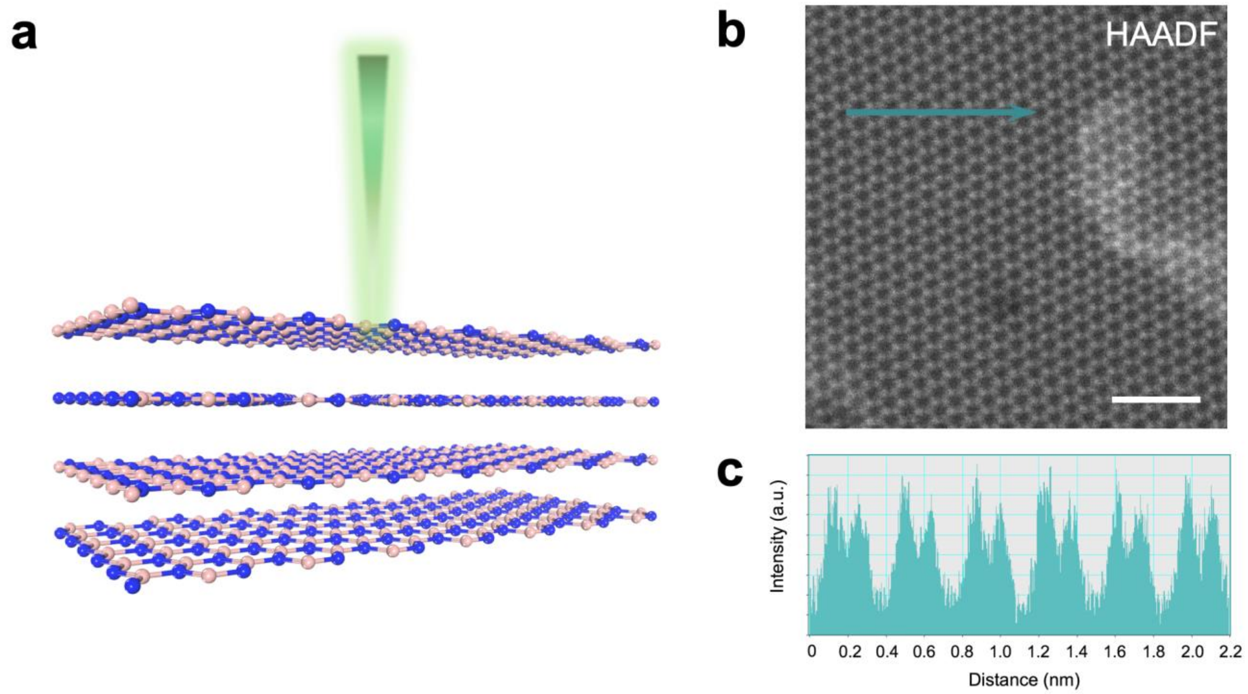

Supplementary Figure 11 | Plane-view STEM measurement for a *h*-BN multilayer. a, Schematic of how the STEM electron beam is applied onto the *h*-BN sample. The electron beam penetrates the *h*-BN along its [0001] crystallographic plane. **b,** Plane-view HAADF image of the multilayer *h*-BN sample. Scale bar, 1 nm. **c,** Profile of intensity corresponding to the green arrow-area noted in **(b)**, shows AA′ stacking structure of the multilayer h-BN crystal. (if it were AA stacked, i.e. N above N and B above B, the two atomic columns would have a very different HAADF intensity; and any staggered layer arrangement would show up as intensity within the hexagons).

Additional characterization of *h*-BN bubbles

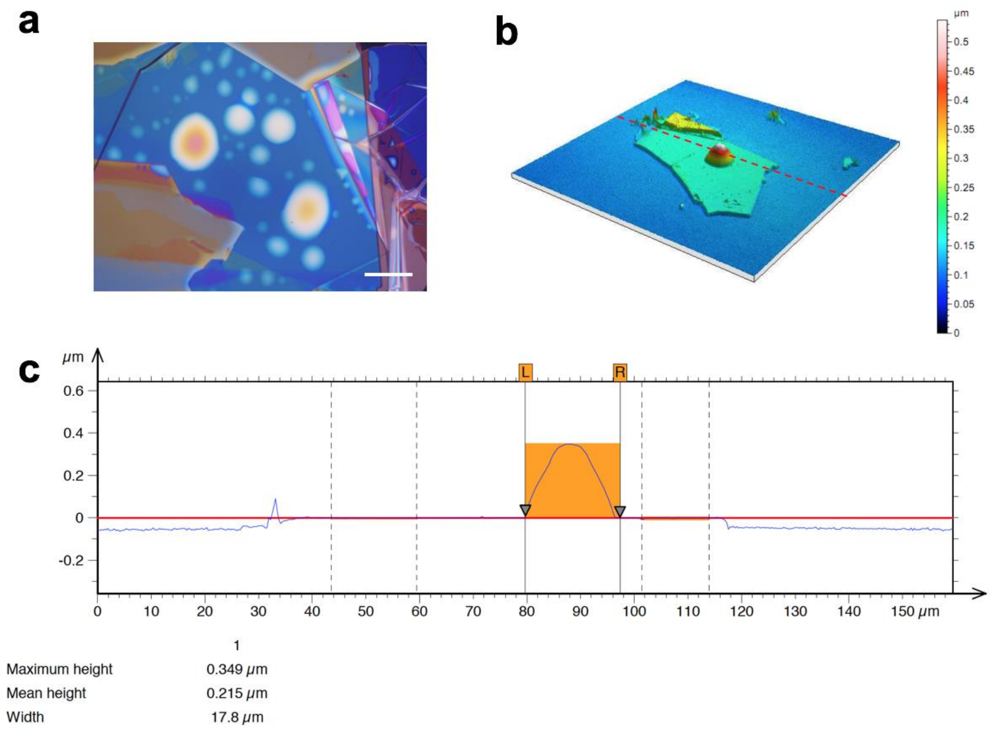

Supplementary Figure 12 | Large *h*-BN bubbles obtained after a 500-minute H-plasma treatment. a, Optical image of the bubbles produced on *h*-BN after a 500-minutetreatment with H-plasma. Scale bar: 20 µm. **b**, Topographic image of a hydrogen bubble (~17.8 µm in diameter) on *h*-BN captured by 3D laser confocal microscopy (NanoFocus usurf). **c**, Height profile along the red dashed line shown in **(b)**, revealing a height of 349 nm at the peak of the bubble.

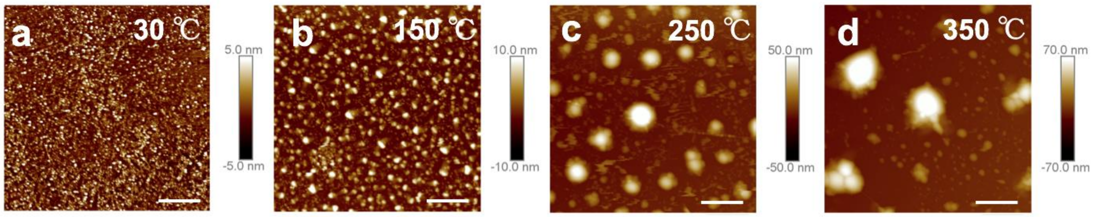

Supplementary Figure 13 | AFM images of hydrogen bubbles after H-plasma treatment. The sample temperature was set at **a**, 30 °C, **b**, 150 °C, **c**, 250 °C and **d**, 350 °C. Scale bars: 2 µm.

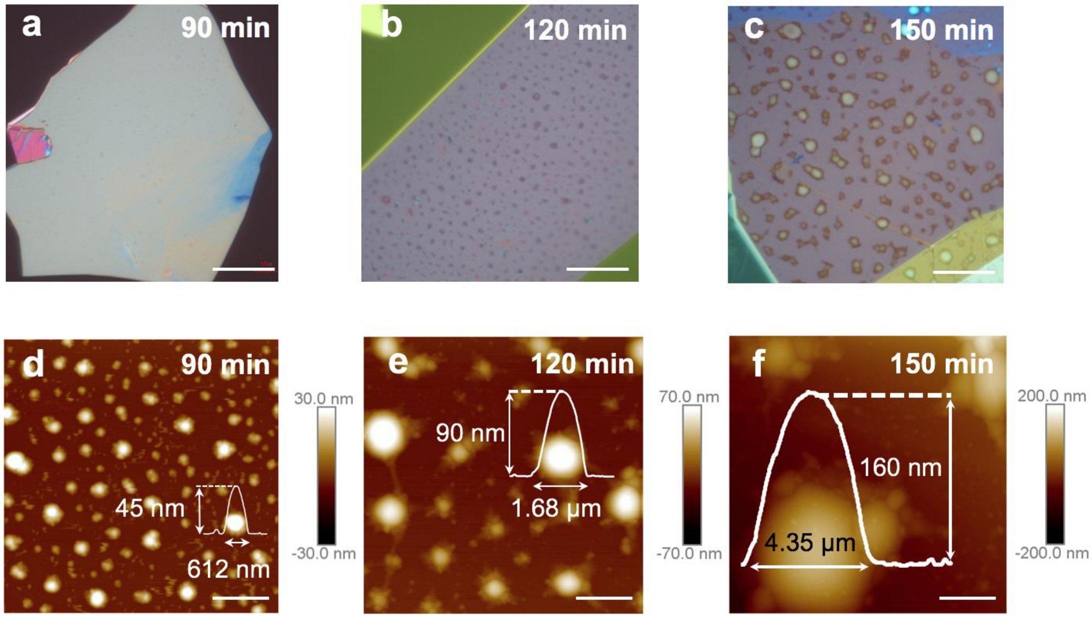

Supplementary Figure 14 | Influence of the H-plasma treatment duration on the bubble dimensions. a-c, Optical images of *h*-BN flakes obtained after H-plasma treatment for 90, 120 and 150 minutes, respectively. Scale bars: 20 μm; **d-f**, AFM height images of the samples shown in **(a-c)**, respectively. The profiles in **(d-f)** give detailed information about the height and diameter of the selected bubbles. Scale bars: 2 μm.

Stability study of hydrogen bubbles on *h*-BN

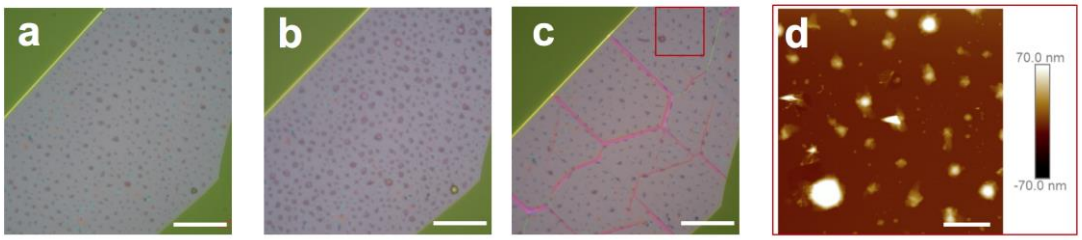

Supplementary Figure 15 | Thermal stability study of *h*-BN bubbles with trapped hydrogen. a, Optical image of *h*-BN bubbles produced by a 120-minute H-plasma (100 W) treatment at 350 °C; the image was collected at a sample temperature of ~30 °C. **b**, Optical image of the same *h*-BN flake taken at 300 ℃ on a heating stage under ambient air. The optical image shows swelling of the bubbles. **c**, Optical image of the same *h*-BN flake after annealing in an Ar/O_2 atmosphere at 800 °C; most bubbles are still visible after the high-temperature treatment, while *h*-BN wrinkles appear on the *h*-BN surface. **d**, AFM height image of the area marked by the red box in **(c)** showing that most bubbles survived after high-temperature annealing in an Ar/O_2 atmosphere. Scale bars: **(a-c)** 20 μm and **(d)** 4 μm.

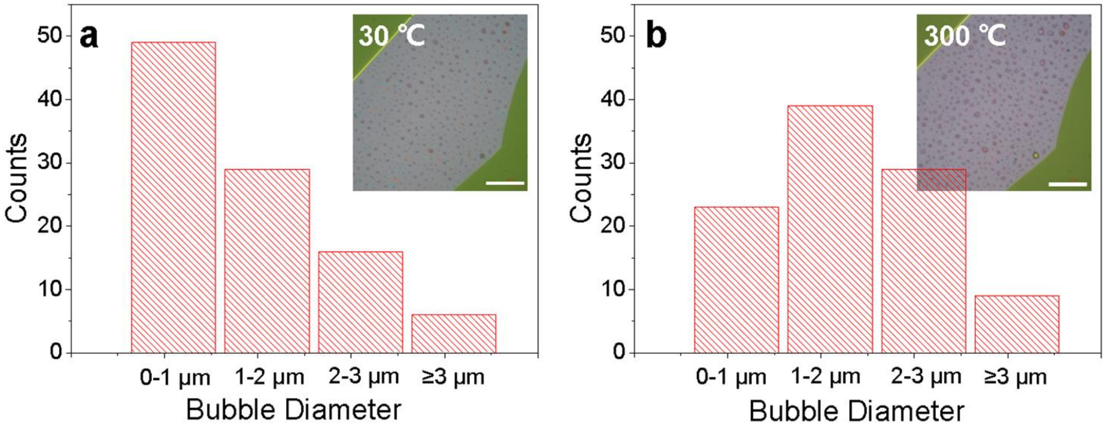

Supplementary Figure 16 | Distribution of the bubble dimensions at different heating temperatures. **a**, Statistics of the bubble diameter at 30 °C; **b**, Size distribution of the *h*-BN microbubbles at 300 °C. The insets in **(a, b)** show the optical images of the same *h*-BN flakes as those provided in Supplementary Fig. 15. The scale bars in the insets represent 20 μm.

An *h*-BN sample with bubbles was placed on a heating stage, where the temperature could be controlled from 30 to 300 °C. An optical microscope (Eclipse LV150, Nikon) was used to record variations in the bubble morphology for the same *h*-BN flake at both 30 °C and 300 °C. Supplementary Fig. 16a exhibits the diameter distribution of the bubbles at 30 °C, while Supplementary Fig. 16b shows the distribution of the bubble diameters on the same *h*-BN flake at 300 °C. As shown in Supplementary Fig. 16, the bubble diameters were clearly enhanced when the substrate temperature was increased from 30 °C to 300 °C. The enhancement of the bubble size reveals that the evolution in the bubble size during the heating process was dominated by the expansion of hydrogen gas, which can be attributed to the fact that the expansion of hydrogen molecules overcomes the fierce competition with the shrinkage of the *h*-BN lattice during heating of the substrate. We also noticed that the bubbles recovered the original dimensions after the substrate cooled to room temperature.

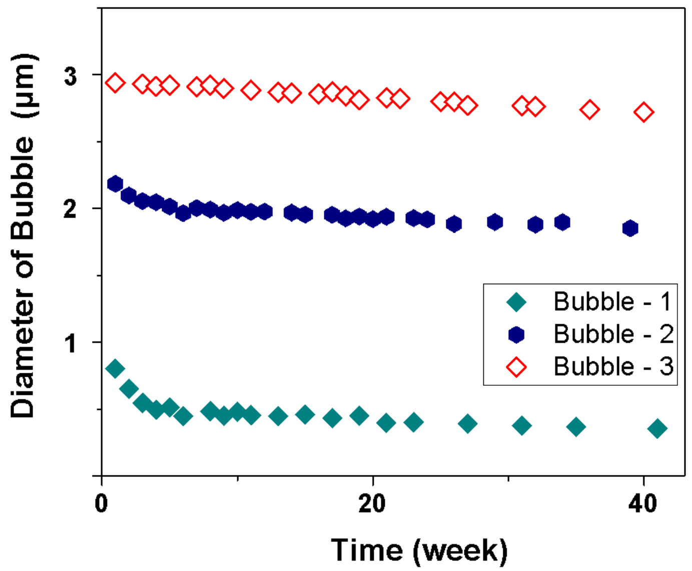

Supplementary Figure 17 | Stability study of hydrogen bubbles on *h*-BN. Geometric evolution of hydrogen bubbles with different dimensions under ambient conditions. This experiment is conducted by measuring the diameter-variation of bubbles on *h*-BN (type-851) with an optical microscope.

The bubbles were firstly subjected to a test lasting ~40 weeks to examine the permeability of the pressured *h*-BN bubbles filled with hydrogen molecules. Bubbles with different diameters (~1, 2, and 3 μm) were tested, and the samples were measured by AFM. The geometric evolutions of the hydrogen bubbles with different dimensions are plotted in Supplementary Fig. 17. Some pronounced tendencies of leakage are observed in the beginning period of the test for the bubbles with diameters of ~1 μm and ~2 μm but not for the bubble ~3 μm in diameter, indicating a higher leakage rate for the bubbles with relatively small diameters in the first 8 weeks. The reason for this difference may be that smaller bubbles (~1 and 2 μm) usually have a much higher inner pressure than large bubble (~3 μm) at the very beginning. A slight decrease in the dimension of the large bubble is also observed in the long-term test, with a decrease in the diameter from ~2.94 μm to ~2.72 μm after 40 weeks of hydrogen storage under ambient conditions. Furthermore, the diameters of all the bubbles tended to be stable after ~30 weeks.

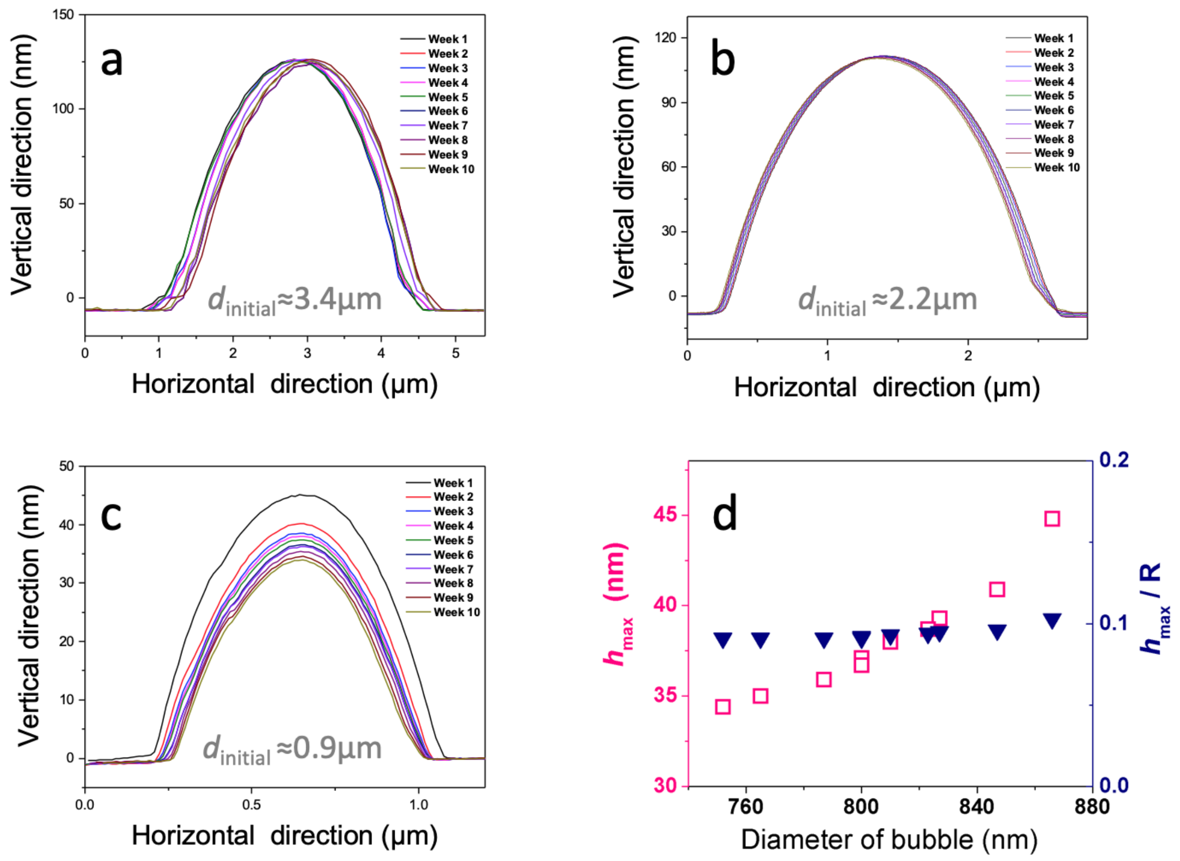

Supplementary Figure 18 | Stability study of the hydrogen bubbles on *h*-BN by cross-sectional profiles of AFM. a, A 10-week tracking of the AFM cross-sectional profile of the bubble with a diameter of 3.4 µm. **b,** A 10-week tracking of the AFM cross-sectional profile of the bubble with a diameter of 2.2 µm. **c,** A 10-week tracking of the AFM cross-sectional profile of the bubble with a diameter of 0.9 µm. **d,** Statistics of the aspect ratio (h_{max}/R) and the height (h_{max}) of the bubble presented in **(c)**

To verify the shrinking-tendency statistics presented in Supplementary Fig. 17, we re-find three new *h*-BN (type-851) bubbles which are similar in dimensions to the former selected bubbles displayed in Supplementary Fig. 17 and re-measure these three bubbles within 10 weeks for about once a week. The results showed a little bit different from our former conclusion. Both the bubbles with a diameter of 3+ µm and 2+ µm are almost no changed in this 10-week period (Supplementary Fig. 18a and b) while bubbles with a diameter of ~1µm do have some shrinkage in this duration (Supplementary Fig. 18c). We also do the statistics of the aspect ratio (h_{max}/R) and the height (h_{max}) of the bubble presented in Supplementary Fig. 18c (Supplementary Fig. 18d), showing that the aspect ratio has almost no change in this duration.

The AFM image, Supplementary Fig. 19, showing that a bubble inside a ruptured outer bubble. We realize that gas molecules in *h*-BN bubbles usually have a distribution in the intervals of different layers rather than accumulate in the gap of top 2 layers, which means that larger bubbles are more likely to have a deeper storage of hydrogen than smaller bubbles. We speculate that the latent reason which is responsible for the leakage of small bubbles could be that they are not

generated as deep as large bubbles by atomic hydrogen and the gas molecules are accumulated at the superficial intervals (van der Waals gaps) where some defects on the surface are more easily to result in the leakage. However, for some larger bubbles, the gas molecules may distribute in many more gaps of the layered material, in which condition the leakage of some top superficial intervals could be neglected.

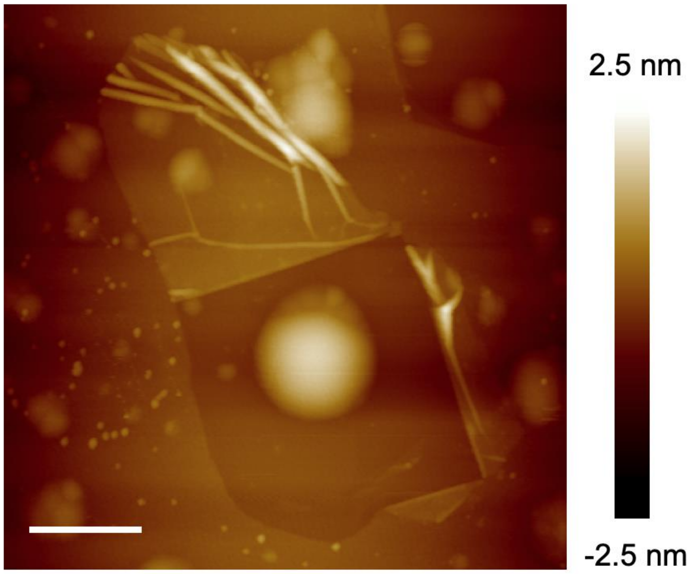

Supplementary Figure 19 | a bubble inside a ruptured outer bubble. Scale bar: 400 nm.

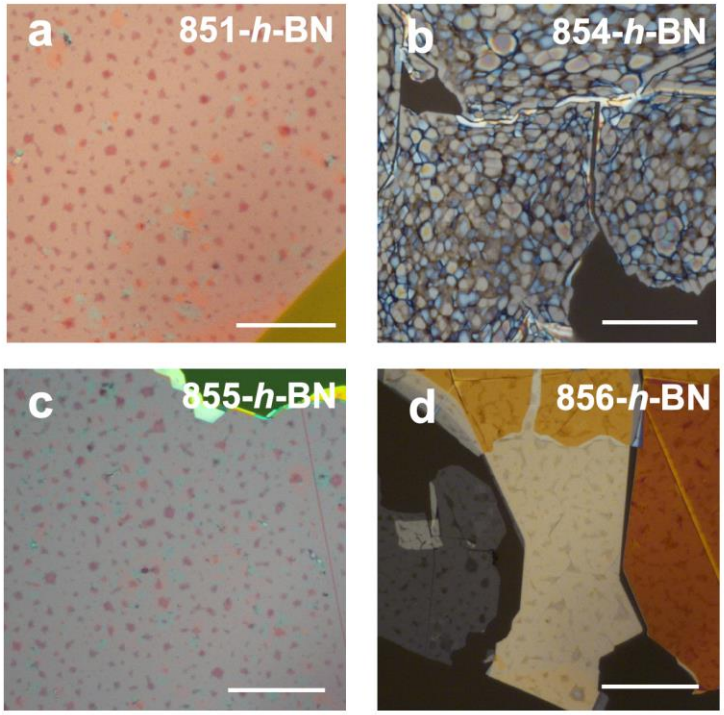

Supplementary Figure 20 | Evaluation of the structural quality of *h*-BN flakes via H-plasma treatment. Optical images of different *h*-BN samples labeled as: **a**, 851-*h*-BN, **b**, 854-*h*-BN, **c**, 855-*h*-BN and **d**, 856-*h*-BN after treatment with H-plasma for 120 minutes at 350 °C. The power of the plasma generator was set at ~100 W. The *h*-BN samples were treated under the same conditions. The scale bars in **(a-d)** represent 20 μm.

h-BN samples fabricated under 4 different conditions (851-*h*-BN, 854-*h*-BN, 855-*h*-BN, 856-*h*-BN) are subjected to H-plasma treatment simultaneously in the same conditions. The results of hydrogen bubbles formation are given in Supplementary Fig. 20. It is obvious that bubble distribution is different on the *h*-BN samples. We believe it may be related to the crystallinity of *h*-BN samples.

Mechanisms of the plasma driven effect

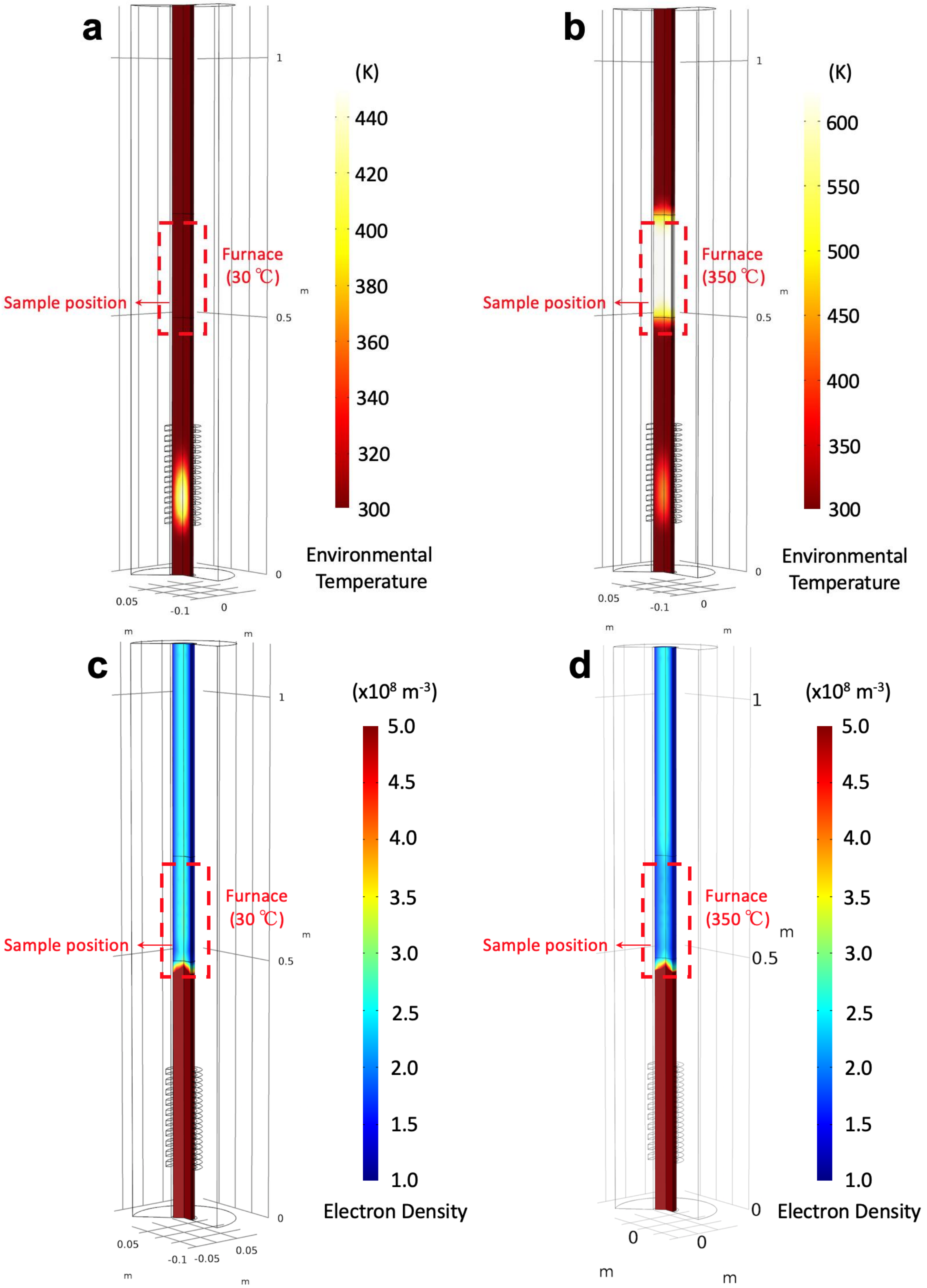

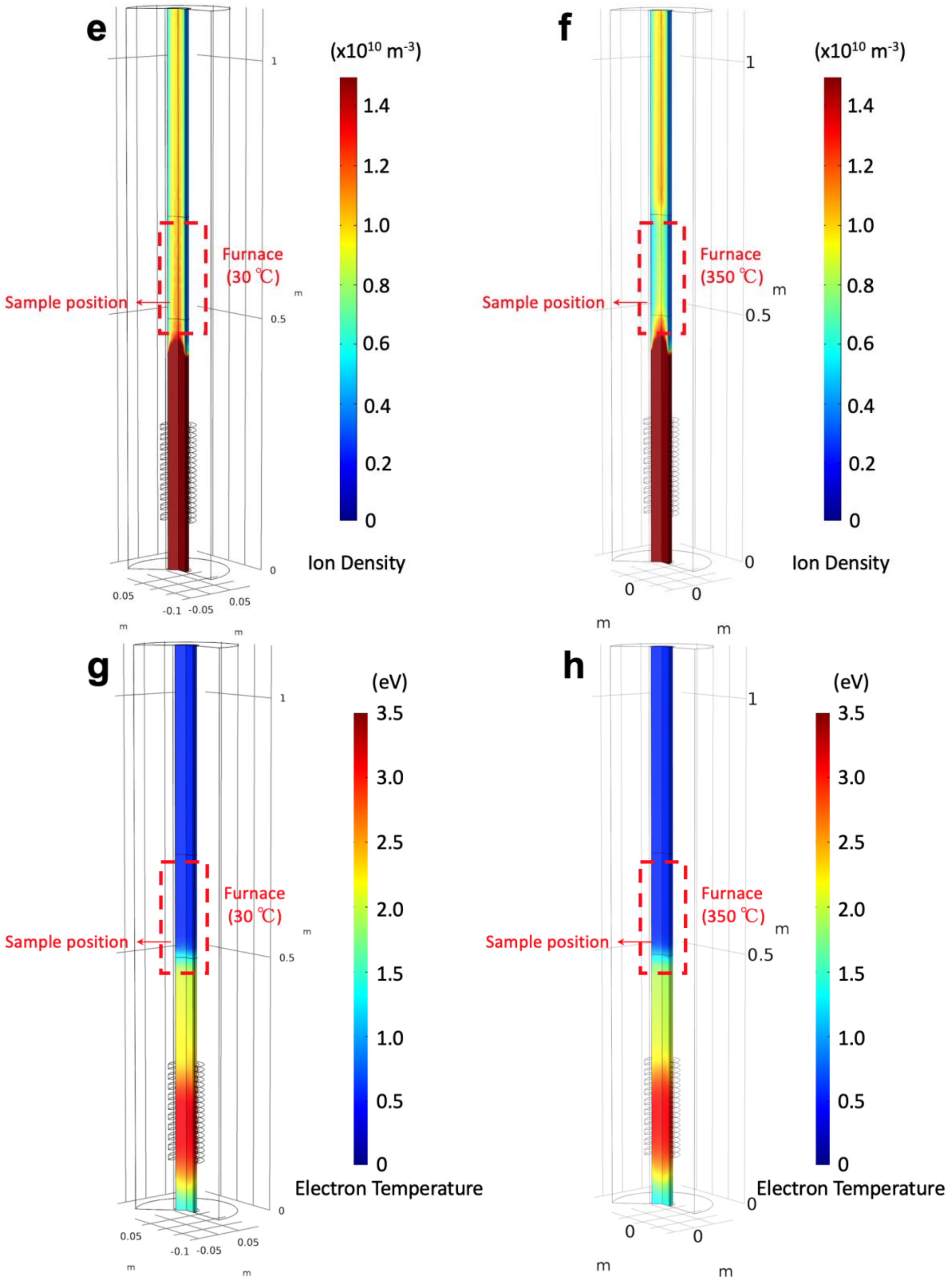

e
(x10^{10} m^{-3})
Furnace
(30 ℃)
Sample position
Ion Density
f
(x10^{10} m^{-3})
Furnace
(350 ℃)
Sample position
Ion Density
g
(eV)
Furnace
(30 ℃)
Sample position
Electron Temperature
h
(eV)
Furnace
(350 ℃)
Sample position
Electron Temperature

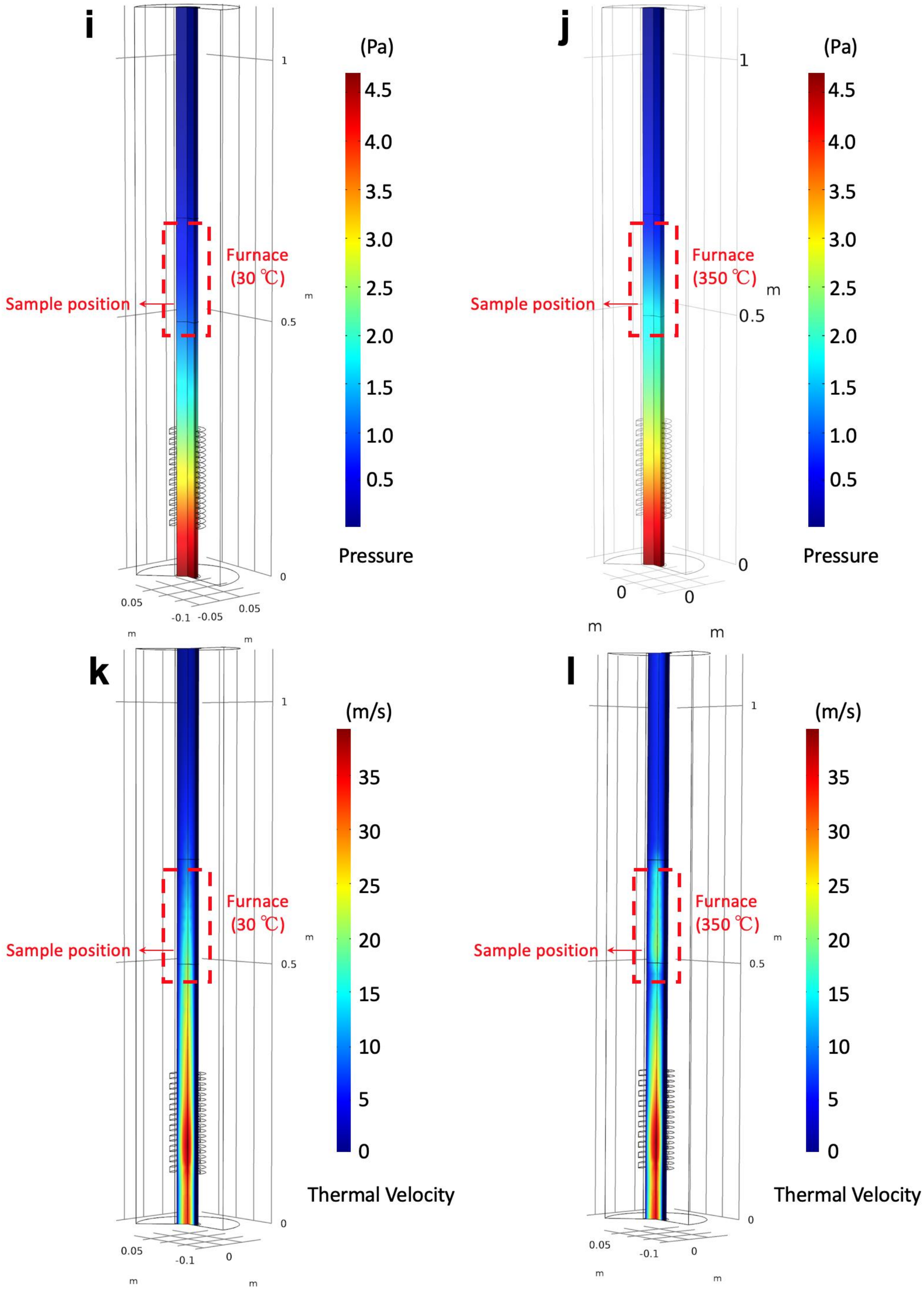
i
j
k
l
(Pa)
4.5
4.0
3.5
3.0
2.5
2.0
1.5
1.0
0.5
Pressure
(m/s)
35
30
25
20
15
10
5
0
Thermal Velocity
Furnace
(30 ℃)
Furnace
(350 ℃)
Sample position
1
0.5
0
m
0.05
-0.1
-0.05
0

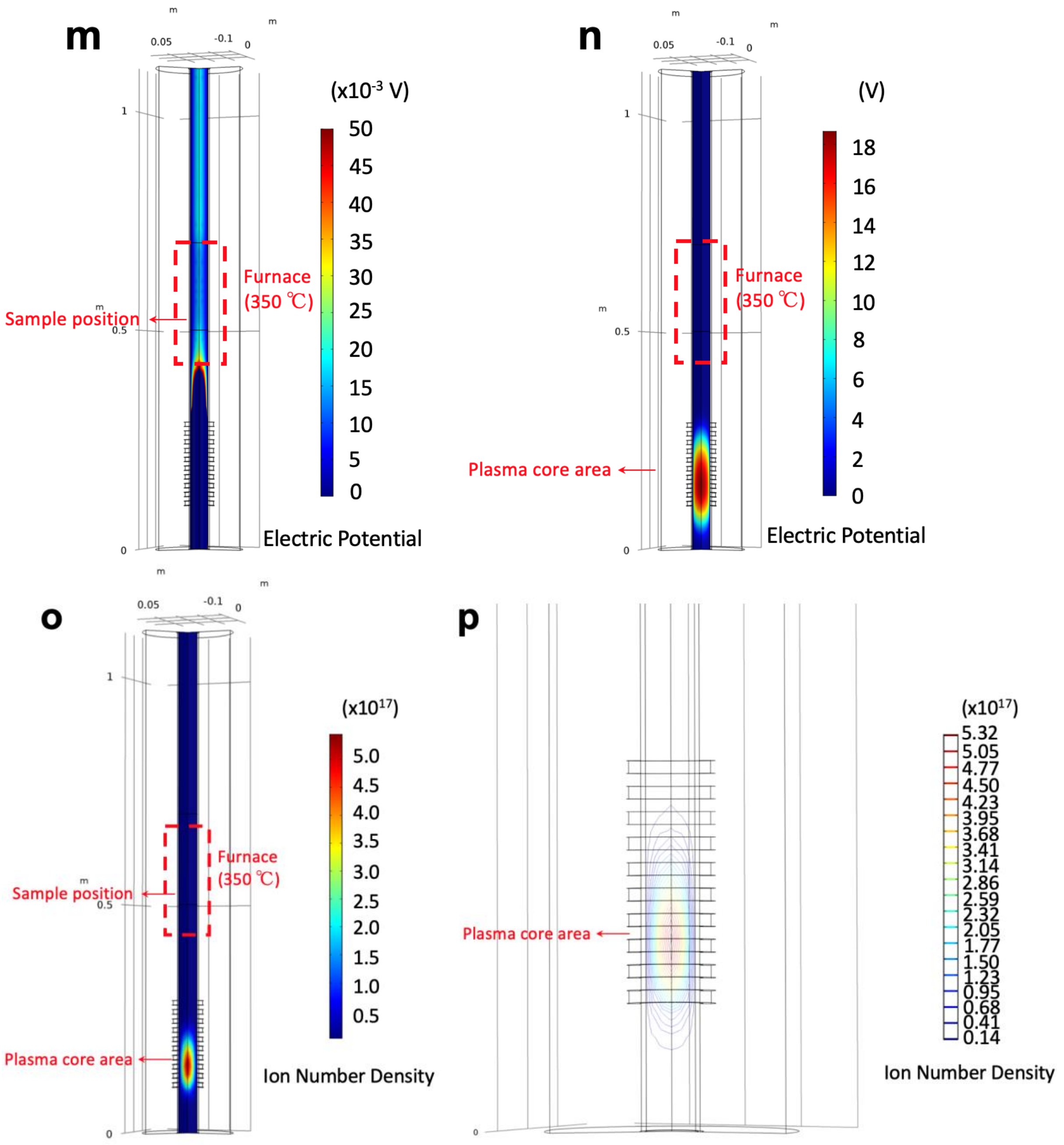
m
(x10-3 V)
50
45
40
35
30
25
20
15
10
5
0
Furnace
(350 ℃)
Sample position
Electric Potential
n
(V)
18
16
14
12
10
8
6
4
2
0
Furnace
(350 ℃)
Plasma core area
Electric Potential
o
(x10¹⁷)
5.0
4.5
4.0
3.5
3.0
2.5
2.0
1.5
1.0
0.5
Furnace
(350 ℃)
Sample position
Plasma core area
Ion Number Density
p
(x10¹⁷)
5.32
5.05
4.77
4.50
4.23
3.95
3.68
3.41
3.14
2.86
2.59
2.32
2.05
1.77
1.50
1.23
0.95
0.68
0.41
0.14
Plasma core area
Ion Number Density

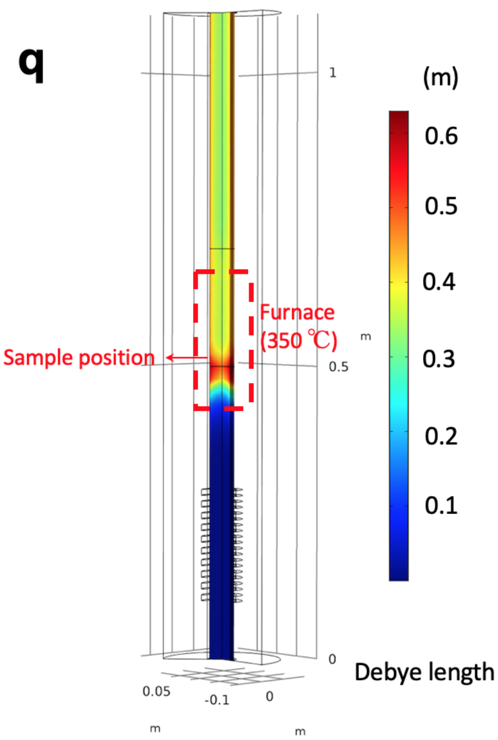

Supplementary Figure 21 |Distribution of different physical parameters in the tube under different conditions. The environmental temperature distribution in the tube when the temperature is set from **a,** 30 ℃ to **b,** 350 ℃ in the furnace marked as a dashed frame. Contrast of electron density in the tube when the furnace temperature is set from **c,** 30 ℃ to **d,** 350 ℃. Contrast of ion density in the tube when the furnace temperature is changed from **e,** 30 ℃ to **f,** 350 ℃. Contrast of electron temperature in the tube when the furnace temperature is set from **g,** 30 ℃ to **h,** 350 ℃. Contrast of pressure in the tube when the furnace temperature is set from **i,** 30 ℃ to **j,** 350 ℃; and Contrast of thermal velocity in the tube when the furnace temperature is changed from **k,** 30 ℃ to **l**, 350 ℃. **m,** Details of the electric potential distribution at the sample position when the furnace temperature is set to 350 ℃. **n,** Details of the electric potential distribution at plasma core area (there is no heater at plasma core region). **o,** Details of the ion number density distribution at the plasma core region. **p,** Zoom-in contour of ion number density at the plasma core region. **q,** Distribution of Debye length in the whole tube when the furnace temperature is set at 350 ℃.

We try to do detail analysis about plasma driven kinetic mechanisms. Supplementary Fig. 21 shows the collateral effects of some different physical parameters result from the variation of the sample temperature by Comsol simulations. The simulations exhibit some differences of physical distribution in the plasma tube when the environmental temperature at sample position was set to 30 ℃ and 350 ℃ by the furnace (Supplementary Fig. 21a-b). It is obvious that when the environmental temperature increases, the electron density is decreased along with the ion density (Supplementary Fig. 21c-d, e-f), the electron temperature is slightly decreased but almost keep the same (Supplementary Fig. 21g-h), and the pressure and thermal velocity at the sample position are both increased (Supplementary Fig. 21i-j, k-l). From the simulation results, we found that the ion

density decreases while the thermal dynamic velocity of ions increases. This is a possible reason to explain why the density of bubble decreases and the size of bubbles increases when the samples were heated up (See in Fig. 3c in main manuscript). Both Supplementary Fig. 21m and Supplementary Fig. 21n show the distribution of electric potential in the reaction tube from the same simulation. As there is a large change in the magnitude of electric potential between the plasma core region and the sample area, we plot the electric distribution of these two areas separately in different scale for clarity. Supplementary Fig. 21o presents the ion number density at the plasma core area. To clearly show the ion number density at the plasma core area, we also put a zoom-in contour view in Supplementary Fig. 21p. The distribution of Debye length is also simulated, and presented in Supplementary Fig. 21q. Debye length reaches its maximum at the sample position (~ 0.5 m). This value is much higher than the diameter of our reaction tube (0.042 m). It indicates that the quasi-neutrality at the sample position is destroyed. This interprets why the electron density is 2 orders lower than the ion density at the sample position in our pervious simulation. Moreover, a higher value of Debye length in the sample position can be achieved if the heated furnace is set at 350 ℃. Normally, Debye length depends on density of ion number and environmental temperature. Debye length increases when ion number density decreases or when the environmental temperature increases.

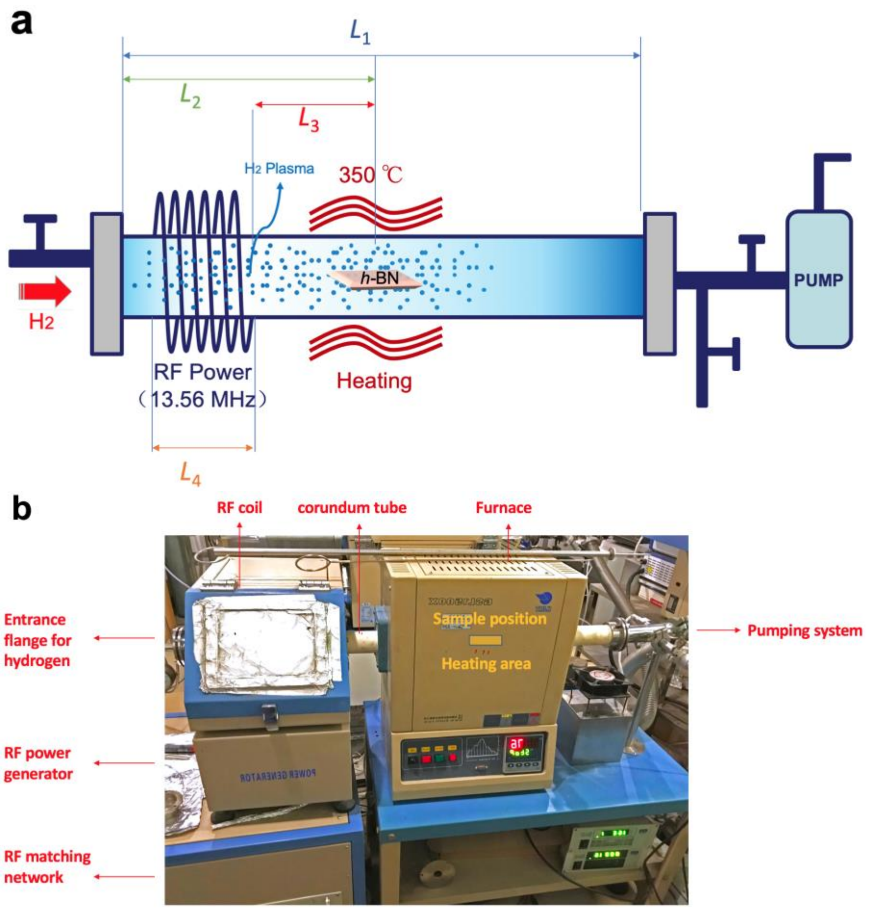

c

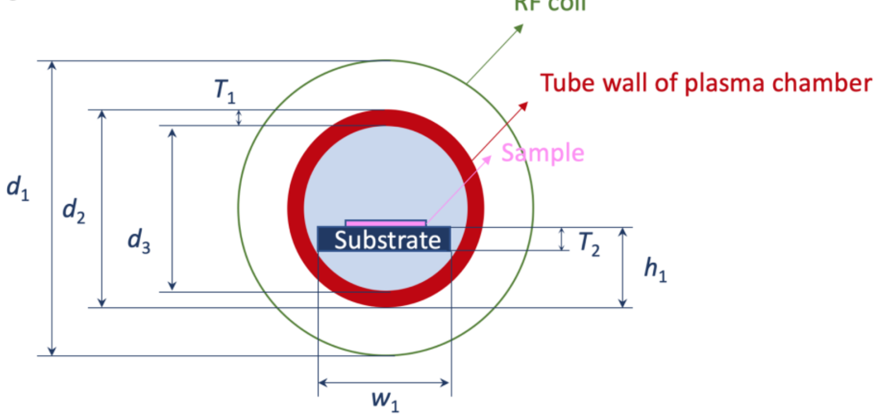

d

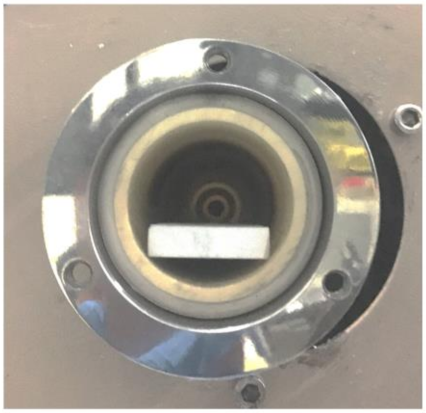

Supplementary Figure 22 | Setup used to fabricate hydrogen bubbles on *h*-BN. **a,** Side view of the furnace for bubble fabrication, where L_1 (110 cm) is the length of the plasma tube, L_2 (60 cm) is the distance from the entrance to the *h*-BN sample, L_3 (30 cm) is the horizontal distance between the RF coil and the *h*-BN sample and L_4 (20 cm) represents the length of the RF coil. **b,** Lateral photograph of the Plasma system corresponding to **(a)**, captions in the image shows different functional units of the plasma system. **c,** Cross-sectional view of the plasma chamber system for bubble fabrication, where d_1 (12 cm) denotes the diameter of the RF coil, d_2 (5 cm) is the outer-wall diameter of the plasma tube, d_3 (4.2 cm) is the inner-wall diameter of the plasma tube, T_1 (0.8 cm) is the thickness of the wall of the plasma tube, T_2 (0.7 cm) is the thickness of the corundum substrate for sample (the *h*-BN sample is exfoliated onto a quartz substrate which is placed on the corundum substrate), w_1 (3.1 cm) is the width of the corundum substrate and finally h_1 (1.9 cm) represents the height of the sample from the bottom of the tube. **d,** Cross-sectional photograph of the entrance of plasma tube and the corundum sample holder.

Study of bubble-formation-distribution at different sample areas in reaction tube:

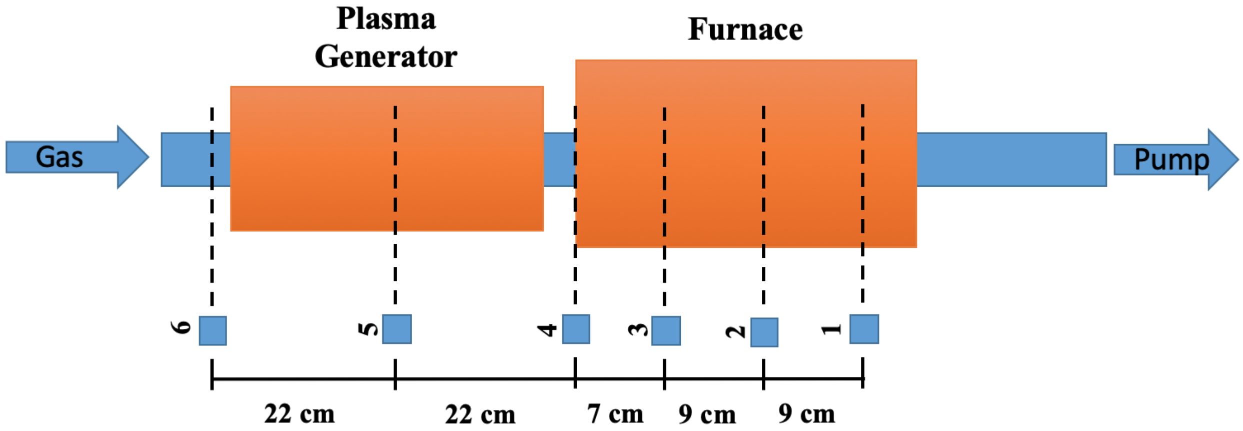

Supplementary Figure 23 | Schematic of different positions to place the *h*-BN samples along the tube. (from Position 1 to 6)

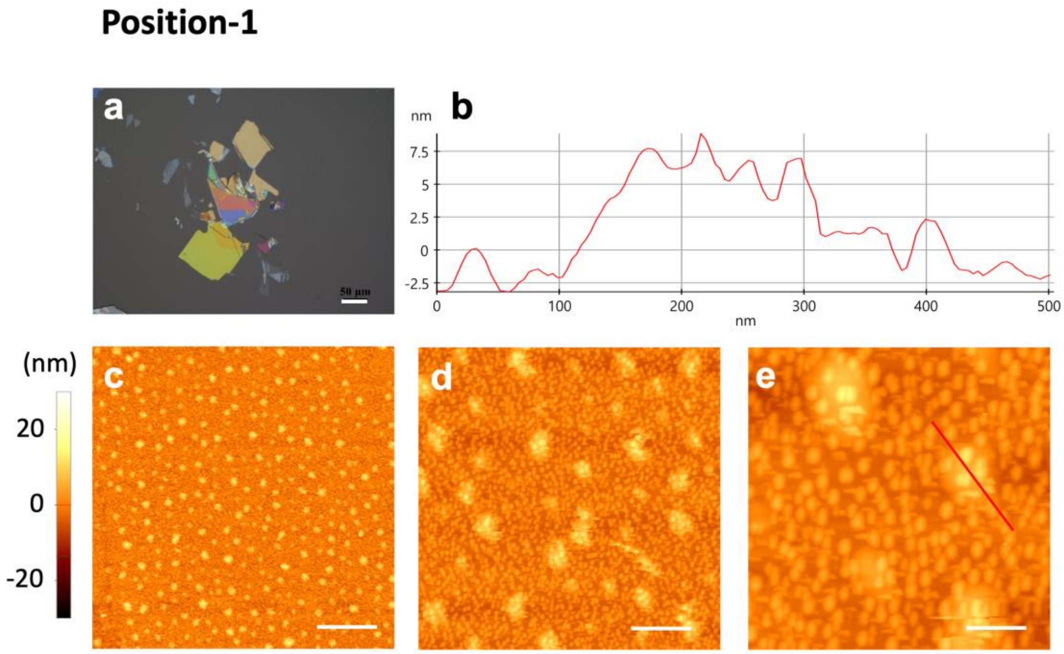

Position-2

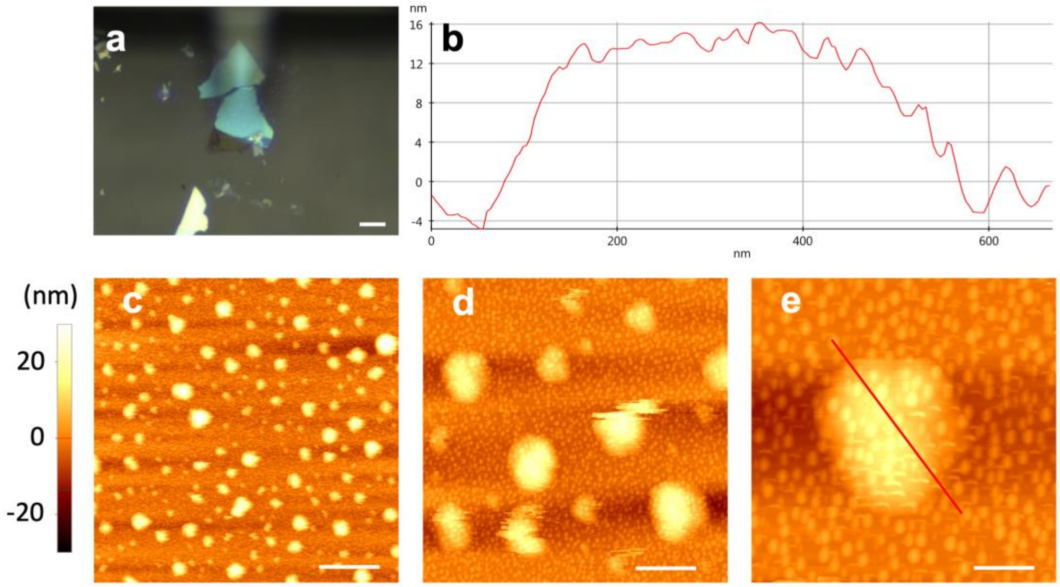

Position-3

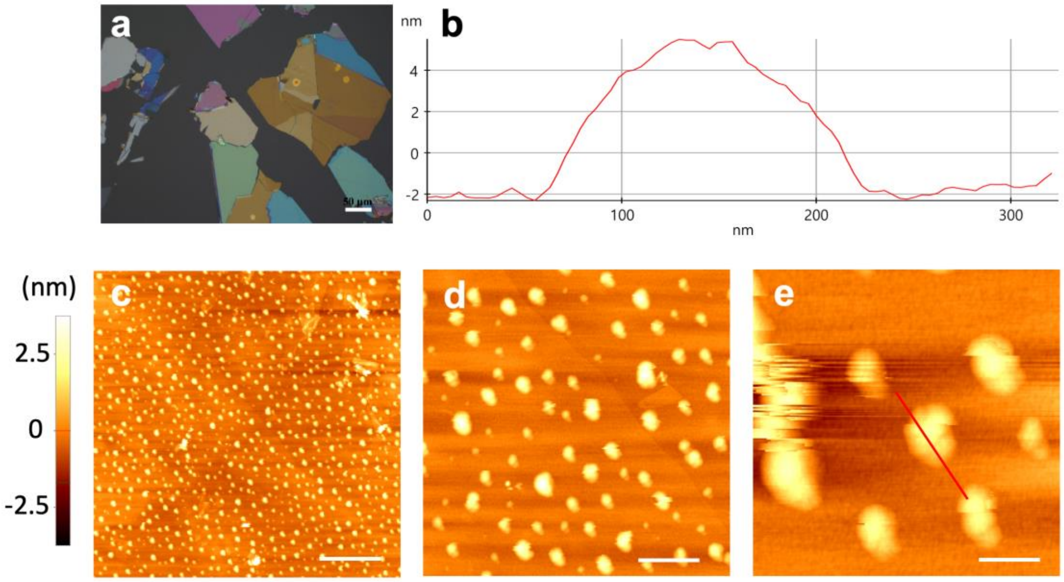

Position-4

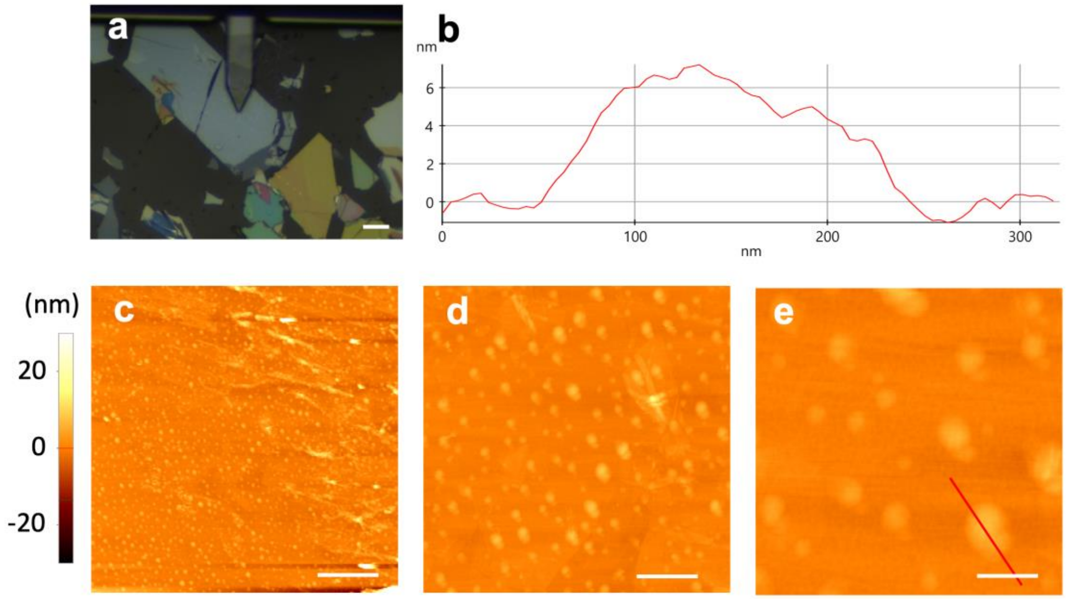

Position-5

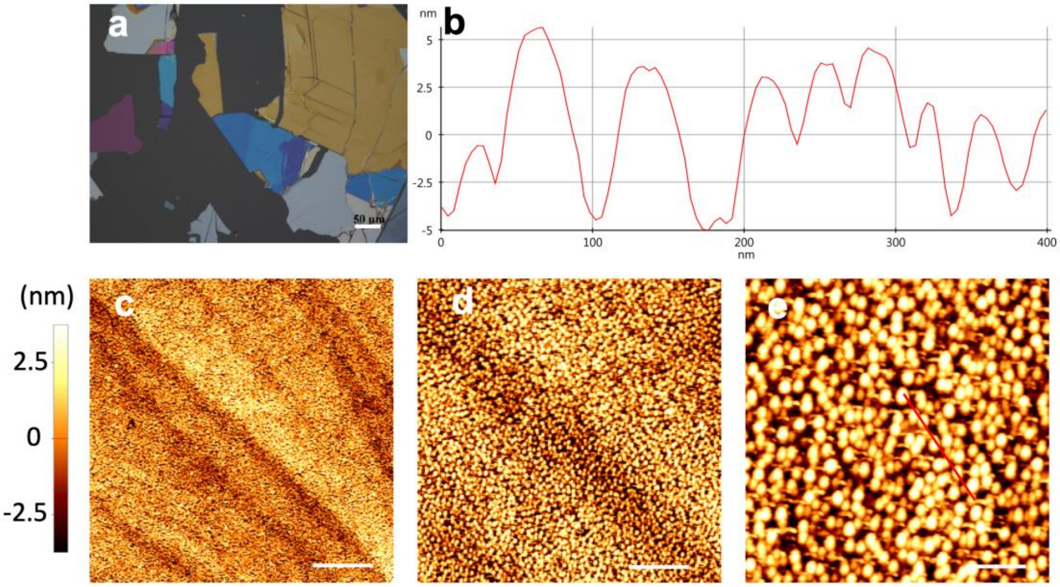

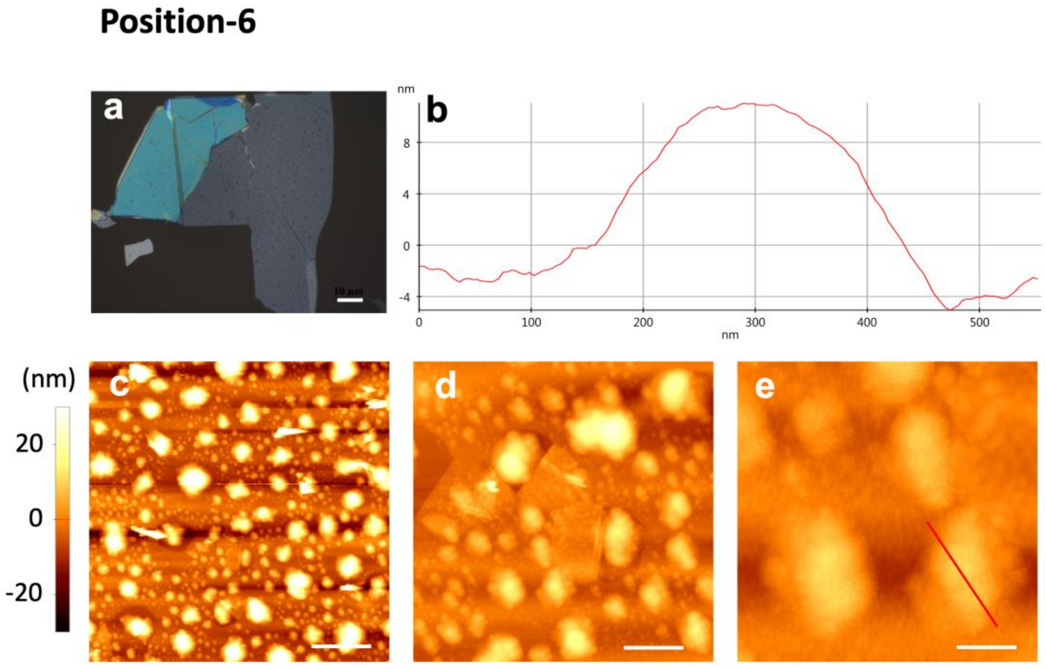

Supplementary Figure 24 | Optical image and AFM topographic image of *h*-BN samples placed from Position 1 to Position 6. For the *h*-BN samples at each position, **a,** The optical image of *h*-BN sample. Scale Bar: 50 μm. **b,** The cross-sectional profile which is corresponding to the red line marked in (e). **c-e,** AFM topographic images in different scan area. Scale Bar, (c): 2 μm, (d): 600 nm, (e): 200 nm.

We placed six *h*-BN samples at Position 1-6 along in the tube as shown in Supplementary Fig. 23 in order to investigate the difference in bubble formation. There are detailed experimental parameters: RF power in 100 W, the center of the furnace at 350 ℃, a H_2 flow in a rate of 3 sccm, a pressure in 3 Pa and a duration of 60 minute for plasma treatment. AFM and optical images of the *h*-BN samples were taken and the detailed results are shown in Supplementary Fig. 24. As shown in Supplementary Fig. 24, the bubbles on *h*-BN sample at Position 2 (furnace center, 350 ℃) and Position-6 (at the front of the RF coil) are much larger than those on other samples while there is almost no big bubble visible on *h*-BN placed at Position 5 (plasma core area near the center of the RF coil) under optical microscope. However, it is found that there are masses of tiny bubbles in a very high density on *h*-BN samples placed at Position 5 by AFM scanning. To understand these phenomena, we carried out the distribution simulation of both the electric potential (Supplementary Fig. 21m-n) and the ion number density (Fig. Supplementary Fig. 21o-p) in the tube. At the center of the RF coil (position 5), the electric potential reaches its maximum but its gradient is much lower than that of the entrance area of RF coil (Position 6). This difference could lead to the distinction of the ion injection quantity on these two samples (the height of bubbles on *h*-BN at Position 5 is averagely $<$ 5 nm. But for sample placed at Position 6, that is $>$ 15 nm). Moreover, the plasma core region at Position 5 also possesses the maximal ion number density, according to Supplementary Fig. 21o-p. And therefore, it is reasonable that the *h*-BN flakes at Position 5 has the highest density of the bubbles among all *h*-BN samples. On the other hand, *h*-

BN samples located at Position 1-4 also show different distributions of the bubble formation. At the center of the furnace, both the height and diameter values of bubbles reach their maximum of ~18 nm and ~600 nm at Position-2 while bubbles on *h*-BN at Position 1,3 and 4 could only be raised to ~7-10 nm high with the average diameter of ~100-300 nm. It is likely that the environmental temperature of 350 ℃ at the center of the furnace offers some additional energy to the ions, which makes them easier to penetrate the *h*-BN mesh and gives rise to the redistribution and size changing of larger bubble formation at Position 2.

Generally, the energy of the accelerated ions is determined by the comprehensive influence from ion density, Debye length and potential gradient in plasma. A higher density of ions may remarkably increase their collision probability with other ions and electrons, and then increase the probability of their recombination to hydrogen molecules. It could markedly shorten the mean free path of ions at the plasma core area and restrict the ions to obtain enough energy for their accelerations. As such, the density of bubbles in the plasma core area should be very high and exhibits relatively small size. Comparatively, ions can get more energy at the sample area due to the lower ion number density and finally give rise to thin but relatively large bubbles. As shown in Supplementary Fig. 24, the experimental results are consistent with our analysis above: Bubbles in the **plasma core area** are small but dense. while bubbles **at other area** show relatively large but thin.

Low temperature AFM measurement of the bubbles

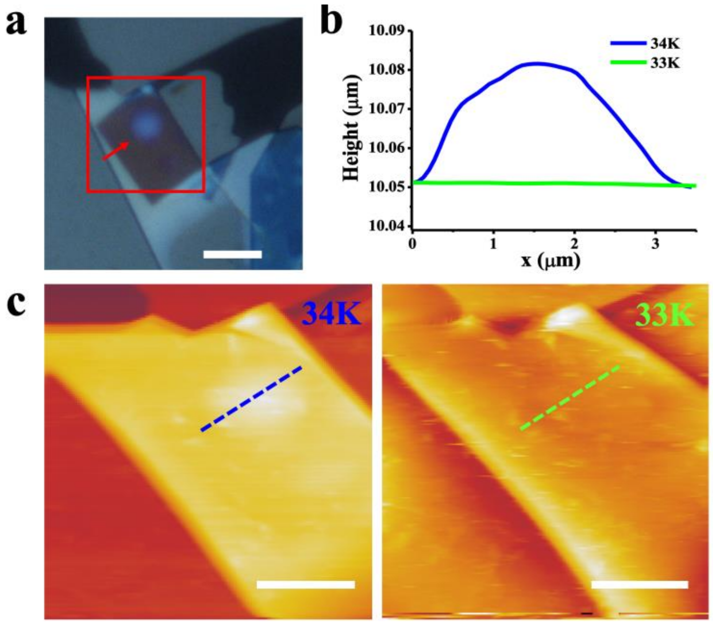

Supplementary Figure 25 | The vanished bubble on *h*-BN surfaces when the temperature is cooling down from 34 K to 33 K. Scale Bar, (a): 5 μm, (c): 3 μm.

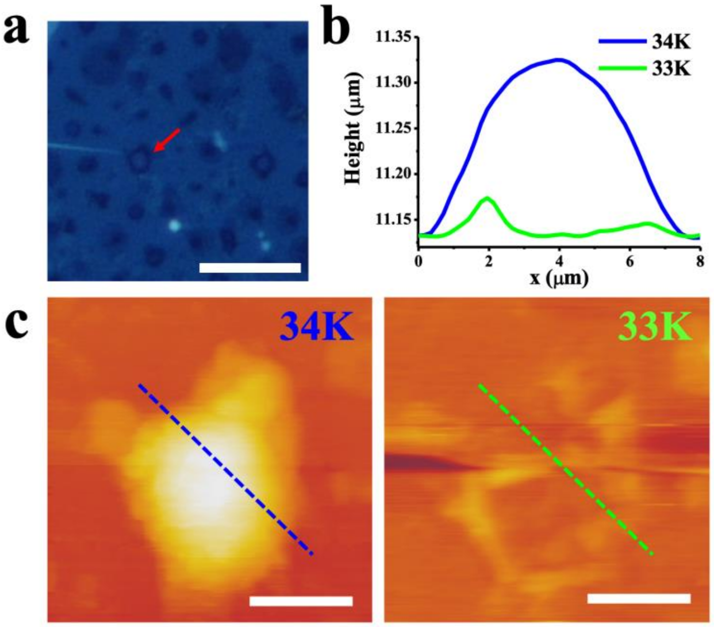

Supplementary Figure 26 | The vanished bubble on *h*-BN surfaces when the temperature is cooling down from 34 K to 33 K. Scale Bar, (a): 20 μm, (c): 3 μm.

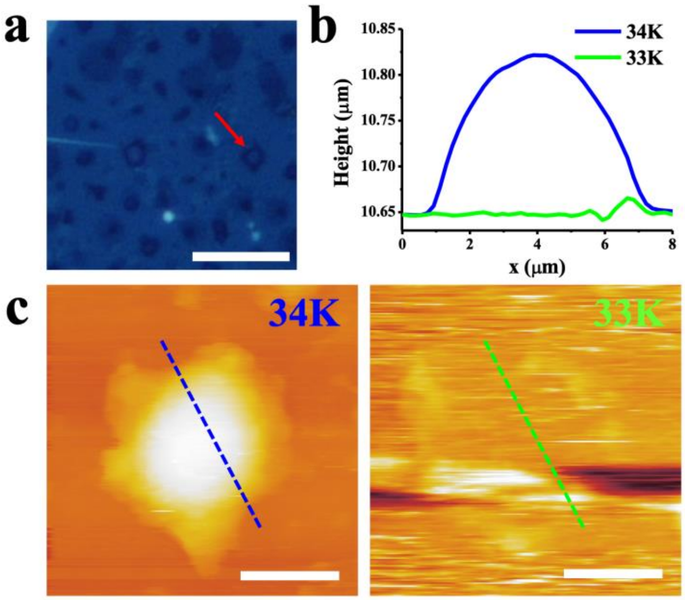

Supplementary Figure 27 | The vanished bubble on *h*-BN surfaces when the temperature is cooling down from 34 K to 33 K. Scale Bar, (a): 20 μm, (c): 3 μm.

During the cooling process from room temperature, we found that bubbles shrink gradually. It can be easily understood by the second law of thermodynamics: $PV/T = constant$, where pressure inside the bubble (P) mainly depends on the elastic modulus of h-BN. It is known that elastic modulus always keeps constant even when the temperature varies. As a result, the volume of the bubble will decrease during the cooling process. The formation of wrinkles on h-BN surfaces shown in Supplementary Fig. 25-27c may be related to the shrinking of the bubbles.

Boiling point and critical point of different gases

	Boiling point	Critical point	
**Hydrogen**	20.271 K	32.938 K,	1.2858 MPa
**Helium**	4.222 K	5.1953 K,	0.22746 MPa
**Nitrogen**	77.355 K	126.192 K,	3.3958 MPa
**Oxygen**	90.188 K	154.581 K,	5.043 MPa
**Argon**	87.302 K	150.687 K,	4.863 MPa
**Methane**	111.65 K	190.6 K,	4.64 MPa

Supplementary Table 2 | The boiling points and liquid-vapor critical points of different gasses adapted from Wikipedia

Temperature dependence of Raman spectra of *h*-BN bubble

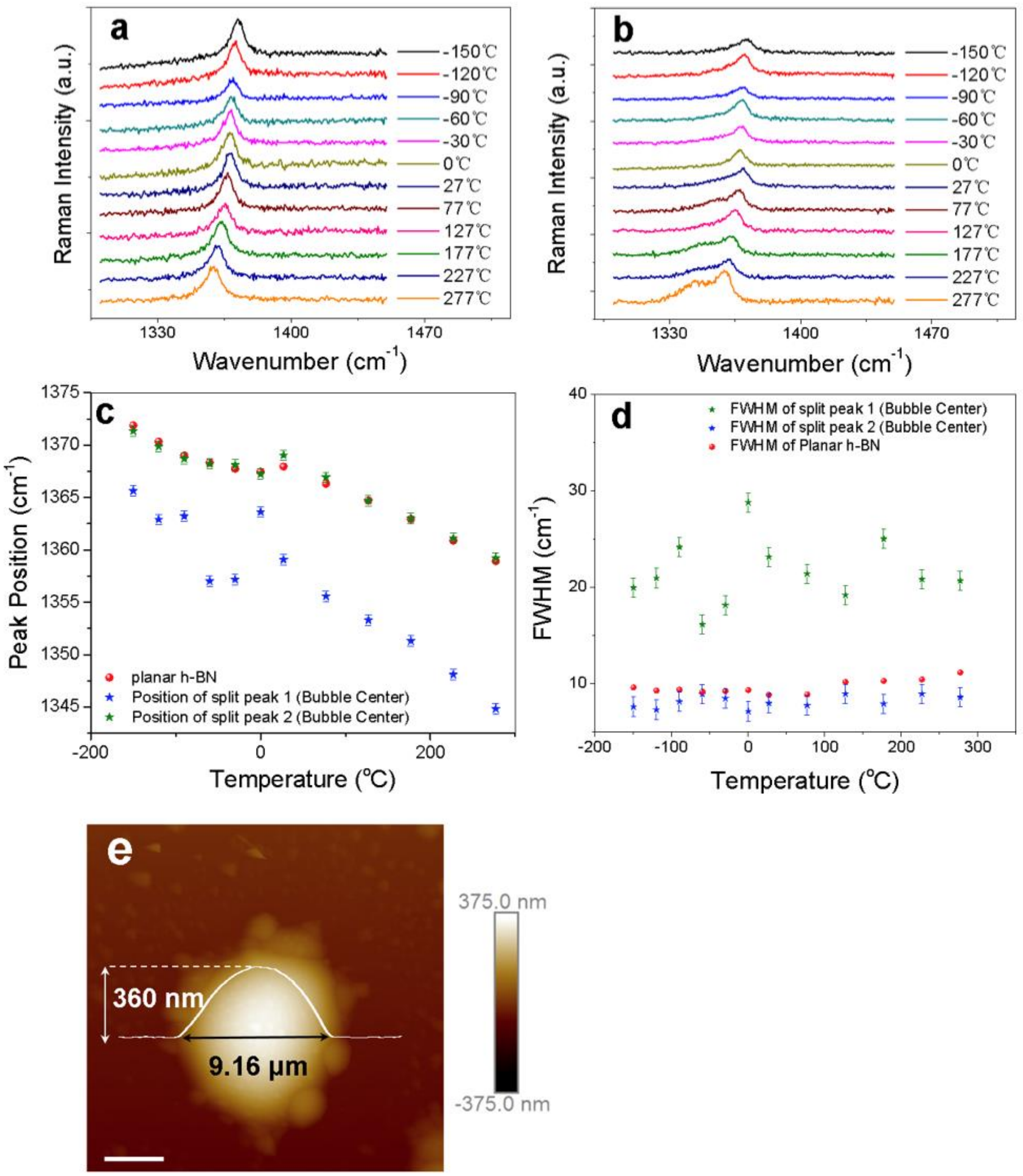

Supplementary Figure 28 | Temperature dependence of Raman spectra taken of an *h*-BN bubble. a, Raman spectra taken of the flat surface of an *h*-BN flake at temperatures ranging from -150 °C to 277 °C. **b**, Raman spectra taken of the center of a bubble on an *h*-BN flake in the temperature range from -150 °C to 277 °C. **c**, E_{2g} peak position distribution from the Raman spectra taken of the flat area (red circles) and the bubble center (both blue and green stars, representing two split E_{2g} peak positions) on the *h*-BN surface with respect to the measurement temperature. **d**, FWHM distribution of the Raman spectra taken of the flat area (red circles) and bubble center (both blue and green stars, representing the FWHM of two split E_{2g} peaks) on the *h*-BN surface in

relation to the measurement temperature. The error bars indicate the standard deviation. **e**, AFM height image of the bubble examined by Raman spectroscopy, revealing a diameter of ~9.16 µm and a height of ~360 nm. Scale bar, 3 µm.

Raman spectroscopy is a powerful tool for investigating the mechanical and thermal properties of *h*-BN. On the flat area of the *h*-BN samples, only one obvious peak appeared in the Raman spectrum in the range from 1310 cm^{-1} to 1450 cm^{-1}, namely, the E_{2g} peak (~1366 cm^{-1}). For the *h*-BN bubbles, the *h*-BN E_{2g} peak broadened and exhibited a slight redshift. We then gradually increased the annealing temperature of the *h*-BN samples from –150 °C to 277 °C and recorded Raman spectra of both the flat area and the bubble of *h*-BN at each temperature point. Supplementary Fig. 28a-b shows the typical Raman spectra of *h*-BN in the flat area and the bubble at incremental temperatures from –150 °C to 277 °C. The E_{2g} peak for the flat area of *h*-BN exhibited an obvious redshift, while both a redshift and a split of the E_{2g} peak were observed for the *h*-BN bubble during the heating process. Supplementary Fig. 28c-d show the evolution of the position and FWHM of the peaks.
